# The assessment of general movements in term and late-preterm infants diagnosed with neonatal encephalopathy, as a predictive tool of cerebral palsy by 2 years of age—a scoping review

**DOI:** 10.1186/s13643-021-01765-8

**Published:** 2021-08-12

**Authors:** Judy Seesahai, Maureen Luther, Paige Terrien Church, Patricia Maddalena, Elizabeth Asztalos, Thomas Rotter, Rudaina Banihani

**Affiliations:** 1 Sunnybrook Health Sciences Centre, University of Toronto, Toronto, ON Canada; 2 Department of Pediatrics, Division of Neonatal-Perinatal Medicine, University of Toronto, Toronto, Canada; 3 Queen’s University, Kingston, ON Canada; 4 Newborn & Developmental Paediatrics, Sunnybrook Health Science Centre, 2075, Bayview Ave., Toronto, ON M4N 3M5 Canada

**Keywords:** Neonatal encephalopathy, General Movement Assessment, Prechtl, Hypoxia–ischemia encephalopathy, Cerebral palsy, Infants, Neonates, Term babies, Preterm babies, Motor development

## Abstract

**Background:**

The General Movements Assessment is a non-invasive and cost-effective tool with demonstrated reliability for identifying infants at risk for cerebral palsy. Early detection of cerebral palsy allows for the implementation of early intervention and is associated with better functional outcomes. No review to date has summarized the utility of the General Movements Assessment to predict cerebral palsy in term and late-preterm infants diagnosed with neonatal encephalopathy.

**Methods:**

We conducted a scoping review involving infants born greater than or equal to 34 weeks gestational age to identify all available evidence and delineate research gaps. We extracted data on sensitivity, specificity, and positive and negative predictive values and described the strengths and limitations of the results. We searched five databases (MEDLINE, Embase, PsychINFO, Scopus, and CINAHL) and the General Movements Trust website. Two reviewers conducted all screening and data extraction independently. The articles were categorized according to key findings, and a critical appraisal was performed.

**Results:**

Only three studies, a cohort and two case series, met all of the inclusion criteria. The total number of participants was 118. None of the final eligible studies included late-preterm neonates. All three studies reported on sensitivity, specificity, and positive predictive and negative predictive values. An abnormal General Movement Assessment at 3–5 months has a high specificity (84.6–98%) for cerebral palsy with a similarly high negative predictive value (84.6–98%) when it was normal. Absent fidgety movements, in particular, are highly specific (96%) for moderate to severe cerebral palsy and carry a high negative predictive value (98%) when normal. In the time period between term and 4–5 months post-term, any cramped synchronized movements had results of 100% sensitivity and variable results for specificity, positive predictive value, and negative predictive value.

**Conclusions:**

A normal General Movements Assessment at 3 months in a term high-risk infant is likely associated with a low risk for moderate/severe cerebral palsy. The finding of cramped synchronized General Movements is a strong predictor for the diagnosis of cerebral palsy by 2 years of age in the term population with neonatal encephalopathy. The deficit of high-quality research limits the applicability, and so the General Movements Assessment should not be used in isolation when assessing this population.

**Systematic review registration:**

Title registration with Joanna Briggs Institute. URL: http://joannabriggswebdev.org/research/registered_titles.aspx.

**Supplementary Information:**

The online version contains supplementary material available at 10.1186/s13643-021-01765-8.

## Background

Cerebral palsy (CP) is defined by the Executive Committee for the Definition of Cerebral Palsy in 2006 as “a group of disorders of the development of movement and posture, causing activity limitation, that are attributed to non-progressive disturbances that occurred in the developing fetal or infant brain” [1]. Cerebral palsy is known to be a potential outcome of perinatal asphyxia, with at-risk neonates presenting with encephalopathy, specifically hypoxic-ischemic encephalopathy (HIE) [2]. The American Academy of Pediatrics and the American College of Obstetrics and Gynecology have outlined specific criteria for the diagnosis of HIE [2], and there is strong evidence for therapeutic hypothermia [3, 4] for those term or late-preterm presenting with HIE. Therapeutic hypothermia is shown to improve survival and the 18-month neurodevelopmental outcomes [5], including CP, but it does not completely eradicate the possibility of long-term neurodevelopmental disability [6].

While therapeutic hypothermia can mitigate any injury associated with HIE, early identification and intervention of CP are imperative as there is now strong evidence demonstrating effective tools for the identification and improved functional outcomes associated with a targeted intervention. Prognostication of long-term neurodevelopmental outcomes has utilized a combination of clinical and radiological tools [7, 8]. One tool that has emerged as a strong predictor of neurological integrity is the General Movements Assessment (GMA), developed by Dr. Heinz Prechtl first in 1979 [9, 10]. This assessment describes the repertoire of complex, highly variable, whole-body movements which emerge in the fetus and continue until the first 4 to 5 months of life [11, 12]. Specific patterns exist at set developmental stages (preterm, writhing, and fidgety), and patterns of stereotypy have also been identified that are associated with CP [13, 14]. Various scoring algorithms have been developed with Prechtl and Hadders-Algra [14] being the most reported.

All reviews to date have focused on an amalgamated population of at-risk infants, combining those born preterm as well as those term and late-preterm infants, most recently in two systematic reviews from 2017 by Novak et al. [15] and 2018 by Kwong et al. [16]. There have been another eight reviews completed between 2001 and 2013; of these, seven were systematic reviews [12, 17–22] and one literature review [23]. There are two reviews around the topic of the predictive value of the GMA pending [24, 25], both of which are systematic reviews.

The key characteristics and main findings of the above reviews on GMA are detailed in the protocol for this systematic review [26] and are included in Additional file 1: Table 1. The systematic reviews from 2017 [15] and 2018 [16] looked at a variety of assessments in early infancy utilized to predict CP, including the GMA, the Hammersmith Infant Neurological Examination (HINE) [27], the Movement Assessment of Infants (MAI) [28], and magnetic resonance imaging (MRI). The 2017 review demonstrated that the GMA had the highest sensitivity (98%) for CP [15]. The 2018 review demonstrated a high sensitivity of 93% (95% confidence interval (CI) 86–96) but a low specificity for CP 59% (95% CI 45–71) with the GMA (using Prechtl scoring algorithm) at 6 weeks (writhing phase). Later assessments (10–20 weeks), from the fidgety period (evaluating for the presence and quality of fidgety movements (FMs)), however, demonstrated a better sensitivity of 97% (CI 95% 93–99) and a specificity of 89% (95% CI 83–93) [16]. The conclusion was those later assessments in infancy with the quality of FMs as assessed by GMA had the strongest predictive value for CP.

To date, there are no current reviews that exist describing the predictive value of GMA in a population of near-term or term neonates presenting with encephalopathy. Based on this gap in the literature, we aim to conduct a scoping review on this specific population, as the first priority was to determine the type and extent of available evidence. The key concepts as it relates to the GMA and its use in this and term late-preterm population need to be clarified. Completion of this scoping review will determine the feasibility of a systematic review and meta-analysis.

### Objectives

The primary research question for this review is: What is the published data on the predictive value of the GMA for the diagnosis of CP by 2 to 3 years of age in infants born at term or late-preterm presenting with NE?

The secondary research question is: What is the gap in the literature when the GMA is used to predict CP by 2 years of age in infants born at term or late-preterm presenting with NE?

## Methods

### Study design

A scoping method was chosen for this type of review to fulfill our objective which requires searching and assessing a wide range of research methodologies involving the use of the GMA in CP prediction. A scoping review captured all types of relevant research on the topic in a systematic, transparent, rigorous, and reproducible manner. This scoping review was conducted in accordance with the JBI methodology for scoping reviews [29]. The objectives, inclusion criteria, and methods for this scoping review were detailed in advance and documented in a proposal (included as Additional file 2). The title of our review was registered with JBI. The protocol was published prior to this review [26] (Additional file 1).

Inherent in the nature of the scoping review is the inclusiveness of a wide range of literature, and so, we anticipated the differences in the data quality. Critical appraisal and data synthesis therefore were challenging in terms of conclusive evidence as opposed to that from a systematic review. The scoping review methodology was however especially advantageous to our question as these types of reviews target areas that have not been comprehensively assessed before.

### Eligibility criteria

The participant, concept, context (PCC) framework for scoping reviews was used to define the review focus and is summarized in Table 1 below (see Additional file 1).

**Table 1:** Inclusion and exclusion criteria for the prediction of CP by the GMA in late-preterm and term infants with NE

	Inclusion criteria	Exclusion criteria
Participants	Studies with infants who were: - ≥ 34 0/7 wks GA - Diagnosis of NE - Had a GMA done between birth up to 6 months of life - had an assessment for CP by at least 2 years of age	Studies in which a diagnosis of CP was made after 2–3 years of age Studies with infants born with life-threatening congenital abnormalities, congenital viral infections, an abnormal karyotype, and metabolic disorders Animal studies
Concept	GMA as a predictor of CP by 2 years of age is the main concept	
Context	Studies that reported on: - Infants with NE managed and diagnosed by the standard of care (neurological history and examination) - Studies from all countries that have outcomes reported in the acute neonatal and in the follow-up period by 2 years of age - Studies in the English language only	Text and opinion papers were not considered for inclusion as this is a highly specific and medical topic

*CP* cerebral palsy, *GA* gestational age, *GMA* general movements assessment, *NE* neonatal encephalopathy, *wks* weeks

#### Participants

Our population of interest was those born at ≥ 34 0/7 weeks GA, and we defined this population based on several factors. First, there are already systematic reviews that have consolidated the evidence in the preterm populations for the use of the GMA in the prediction of neurodevelopmental outcomes, but this does not exist for the older gestational age groups [15, 16]. Secondly, the benefit of therapeutic hypothermia for NE caused by HIE is established in infants ≥ 35 weeks GA, and ongoing research considers the extension of therapeutic hypothermia treatment to a GA younger than 35 weeks [4, 5, 30].

We therefore chose to focus on the population of ≥ 34 0/7 weeks GA, firstly to comprehensively consolidate this body of literature. Secondly, we will lay the foundation for future studies looking at the utility of the GMA in the prediction of CP before and after the introduction of therapeutic hypothermia.

#### Concept

The GMA is a non-invasive tool that has been shown to have predictive validity for CP^6^. Traditionally, CP has been diagnosed by 12 to 24 months [1, 31, 32]. We considered a diagnosis by 2 years as by this time, the majority of diagnoses should be made [14]. Given the practicality of obtaining an assessment for CP at precisely less than or equal to 24 months, we have decided to use the time frame of 2–3 years. We appreciate that milder forms of CP may be diagnosed at ages older than 2 years of age. The stability of the diagnosis of CP is also traditionally better at the older ages. Our aim however was to consolidate the literature for the age at which CP is most likely diagnosed [31–36]. Additionally, in term survivors of hypoxic-ischemic brain injury, spastic quadriparesis is the most common type of CP, although athethoid or spastic hemiparesis also occurs which would likely be evident by 2–3 years [37–40].

For the GMA to be used as a predictive tool for CP, we considered studies which measured sensitivity, specificity, positive predictive value, and negative predictive value as detailed in our protocol. These measurements provide the best guidance for this clinical application [41]. Detailed definitions of concepts can be found in Additional file 1: Table 3.

In terms of the type of GMA, we collected studies using any type of GMA although we are aware that based on the difference of the methods, this may need to be interpreted differently. The two commonly used types are the Prechtl [9, 10] and the Hadders-Algra methods [14]. The Hadders-Algra method classifies the GMs as normal (optimal or suboptimal) or abnormal (mildly or definitely) [14]. In the mildly abnormal category, the movements show a lack of variability and complexity, but the FMs are still present. We note that the “mildly abnormal” category in the Hadders-Algra method [14] would be considered normal with FMs present according to the Prechtl method [9, 10]. In the definitely abnormal category, the movements again lack variability and complexity, but there are no FMs and there may be CS movements present. Thus, the abnormal category for the Hadders-Algra method is quite broad [14]. The definitely abnormal category is the only category with the absence of FMs.

This makes it challenging to compare it to the Prechtl method [9, 10] in terms of the GMs at 3 months as the Prechtl method [9, 10] is specific to determining if the FMs are present, absent, or abnormal.

#### Context

We considered the variability in the diagnosis of NE and so chose a baseline of at least a history and neurological examination for the diagnosis. We chose to look at all countries to be able to be as exhaustive as possible in our search.

Studies in the English language only were considered as there is no team member with adequate language skills to translate from any other language.

### Search strategy and databases searched

A search of the literature covered the databases of MEDLINE, Embase, PsychINFO, Scopus, and CINAHL, to be inclusive of medicine, nursing, allied health professions, sociology, psychology, education, and social work. The General Movements Trust website was also searched [42].

The search strategy was phased, firstly created in Ovid MEDLINE using a combination of index terms and keywords around general movements, Prechtl, brain disease, HIE, and perinatal asphyxia. An initial limited search of Ovid MEDLINE, Embase, and PsychINFO was undertaken to identify articles on the topic (see Additional file 3). There were no previous similar reviews identified. The text words contained in the titles and abstracts of relevant articles, and the index terms used to describe the articles from this limited search were then used to develop a more refined full search strategy in the second phase (Additional file 4).

This scoping review considered both experimental and quasi-experimental study designs including randomized controlled trials, non-randomized controlled trials, before and after studies, and interrupted time-series studies. Case reports, case series, case–control and cross-sectional studies, and systematic reviews were considered. Studies published from at least 1970 were included. Prechtl first described GM in 1979, so we chose the date of 1970 to ensure all the related research would be captured [9, 15]. The reference lists of articles were scanned, and experts in the infant developmental field were consulted to identify studies relevant to our topic.

### Study selection

EndNote X9 (Clarivate Analytics, PA, USA) was used for citation collation. Duplicates were removed manually. Covidence (Covidence Systematic Review Software, Veritas Health Innovation, Melbourne, Australia) was used for screening by two independent reviewers (JS and ML). Disagreements were resolved through a third reviewer (RB). The results of the search were reported in a Preferred Reporting Items for Systematic Reviews and Meta-analyses extension for scoping reviews [43].

Ethical approval was not required as this was a scoping review and did not contain information directly identifying patients or content requiring patient consent. We conducted our bibliographic database searches between April 30, 2019, and March 30, 2020. The reference lists of all full-text relevant studies that were identified were hand-searched for additional relevant studies. Citations were identified and duplicates removed and screened by two independent reviewers (JS, ML). Relevant studies were identified for full-text review and searched for via Google Scholar, institutional journal access, e-Resources, and databases sites. Any disagreements that arose between the reviewers at each stage of the study selection process were resolved through discussion. A third reviewer (RB) was the final arbitrator for any unresolved disagreements.

From the full texts, articles were selected for further review that met most of the inclusion and exclusion criteria. From these, articles were identified that fully met all the criteria. The results of the search were reported in a Preferred Reporting Items for Systematic Reviews and Meta-analyses (PRISMA-ScR) flow diagram [44]. Each article was independently reviewed and assessed by two of the authors (JS, ML). The data was extracted from articles using a data extraction tool developed by the reviewers. The format for the data extraction tool was modeled after being used by Kwong et al. in their 2018 review [16] (see Additional file 6). This decision was taken as that review aligned well with our study in describing the predictive ability of GM for later CP, although not specifically to our population.

The distribution of the studies was determined by year of publication, as well as country of origin. These were important contextual factors as the older studies and the physical and human resources of each country may have posed limiting factors. A major consideration could have been the ability to obtain a representative portion of the population which affects the generalizability of the results. The study characteristics included information such as gender, GA, birth weight, and numbers of term and late-preterm neonates in the sample as well as the overall sample size, the number of neonates diagnosed with NE, and the number of cases of CP.

## Results

**Fig. 1:**
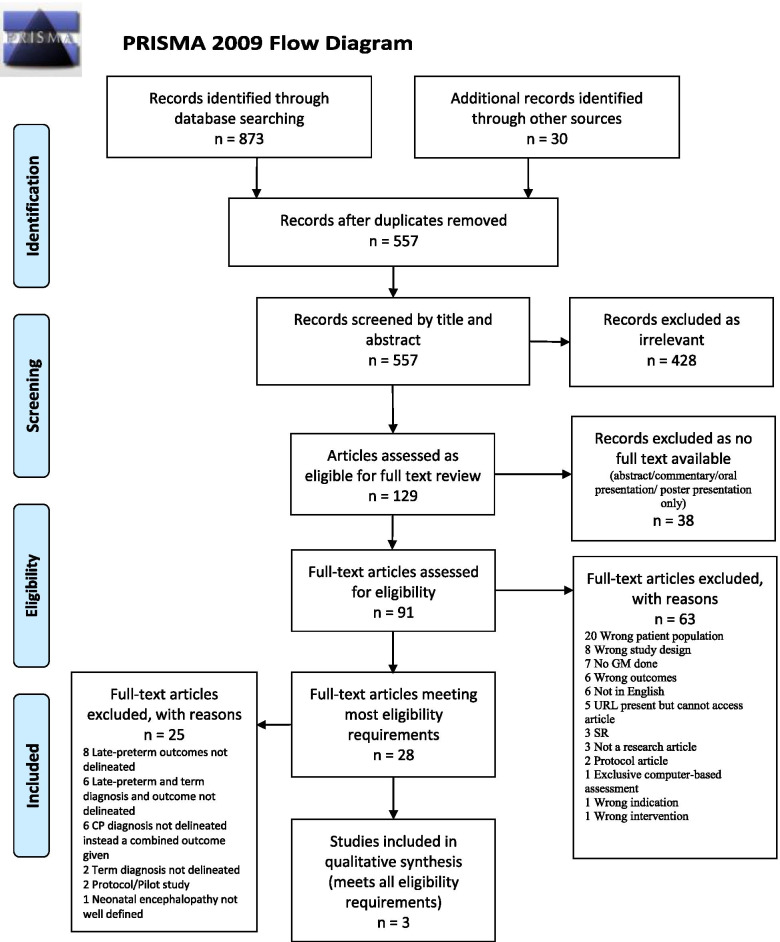
Flow chart for the selection of articles at different phases of the scoping review

Following the searches, 903 citations were identified. The results of the search were reported here in a flow diagram (Fig. 1), adapted from the PRISMA-ScR [44] structure.

There were 25 studies [13, 45–68] that did not meet the full inclusion criteria but contained important information with regard to our topic. We placed these in an exclusion table (Additional file 4: Table 4 and 5). Of these 25 studies, 20 [13, 45–47, 49–52, 54, 55, 57–66] included late-preterm and term infants but did not delineate them as a specific group as it relates to their diagnosis of NE and their CP outcomes and when using GMA as a predictive tool. Additional file 4: Table 4 presents the summary of the characteristics of the excluded studies. There was a wide variety in their key characteristics. We summarized these characteristics here. These studies had a wide date range from 1997 to 2021. They were mainly prospective studies [13, 45, 46, 48–50, 52–54, 57, 59, 60, 62–65] (16 of the 25), and the majority used clinical assessments only to identify infants at high risk [46, 49, 50, 52–54, 60, 61, 63–68] (14 of the 25). With regard to the GMA tools used, of the 25 studies, 23 used the Prechtl GMA and the other two used the Hadders-Algra method [48, 65]. In the study by Dekkers et al. [48], all the children with an abnormal score had either CP or another severe developmental delay.

For the age at which CP was diagnosed, 16 of the excluded studies used the same criteria as we did in this study, that is, CP diagnosis by at least 2–3 years [13, 46, 48, 49, 53, 54, 56–61, 63, 64, 66]. For the method of CP diagnosis, a variety of standardized assessments were used, with the most frequent being by Amiel-Tison and Grenier [69] (5 of the 25) [13, 47, 54, 60, 63] and Touwen Infant Neurological Examination (TINE) [70] (5 of the 25) [46, 47, 49, 54, 64] with other assessments [71–81] detailed in Additional file 4: Table 4.

Eight studies either used non-standardized methods or did not clearly state their method [45, 50, 51, 56, 57, 59, 62, 65].

Additional file 4: Table 5 presents the summary of the key findings of these excluded studies and reasons for their exclusion. These findings showed that in high-risk infants, including those with NE, GMA is a strong predictor of CP [45], especially when used in the fidgety period [46, 53, 55, 57, 61, 63, 65, 66]. In 1997, Prechtl et al. [13] demonstrated that movement quality was important. Abnormal quality and absent fidgety movements, in a mixed group of preterm and term infants, predicted neurological abnormalities with a sensitivity of 96%. The majority of these were diagnosed as CP. We see in our results that over time, this result has been repeatedly duplicated showing that CS [49] and absent fidgety [58] GM are highly predictive of CP. The trajectory of the GMA is more important as a predictor of CP [46].

The GMA is more sensitive than the traditional neurological examination [47, 49, 52], and the sensitivity increases with the combined use of other modalities such as electroencephalogram (EEG) [52], neuroimaging [62], HINE [27], and neuroimaging [58].

For these excluded studies, sensitivity values were as high as 100% [45, 46, 49, 54, 57] and specificity similar close to or at 100% [45, 53, 58, 61, 66]. We contacted the authors, Solemani et al. [61] and Goyen et al. [53], of the studies closest to our inclusion criteria. Solemani et al. [61] delineated their populations by NE and by GA, but their outcome was reported as “neurodevelopmental outcomes” and not CP. They reported to us that they did not specifically report CP and so could not be included for us. Similarly, Goyen et al. [53] reported their outcomes not specifically divided for preterm versus term as their aim was to describe the NICU experience. We were unable to include this study in our final count. Both studies, however, reported on the high predictive validity of the Prechtl GMA at 3 months as it relates to neurodevelopmental outcomes at 2–3 years, and the Goyen et al. [53] study specifically for CP at that age. Nine of the excluded studies [13, 52, 55, 58–62, 66] reported on positive predictive value (PPV) and negative predictive value (NPV) with some studies reporting PPV as high as 98% when used in combination with HINE and neuroimaging [58] or 75% with combined with EEG and ERP [66]. Negative predictive value was reported close to 98.31% [66] or at 100% [54]. Themes for the limitations identified by the authors can be summarized as limited external validity due to small population size [48–50, 56, 58, 63, 64, 66] selection bias related to recruitment from high-risk populations [13, 51, 58], and practice variation between sites [45, 54, 57]. The most common reasons for the exclusion of these studies were failure to delineate their participants for the diagnosis of NE, most quoting their participants as high-risk infants, or not delineating their GA into the groups relevant to our questions (late-preterm and term) [45, 49–54, 57, 58, 60, 62–66].

Only three articles, therefore, Ferrari et al. [82] Glass et al. [83], and Prechtl et al. [84], were identified as meeting the selection criteria and were included in the final review. The results of the search were reported here in a flow diagram (Fig. 1). The final studies included one prospective cohort study from the USA [83], and the other two were case series [82, 84] from Italy. The total number of participants was only 118 term neonates (58, 34, and 26 participants); none included late-preterm neonates. Neonatal encephalopathy was reported as a single group by Glass et al. [83] and Ferrari et al^.^ [82] but divided into mild-moderate and severe by Prechtl et al. [84]. The high-risk groups in the cohort study [83] used a combination of clinical diagnosis, EEG, and MRI where possible to identify the NE population while for both case studies, NE was identified by history only. The GMA used by all three studies was Prechtl. Additional file 4: Table 6 presents the characteristics of these three studies. The prospective cohort study was published in 2021 [83] and reflected data collected within the previous 6 years, which would be reflective of the current management practices, especially as they did report on 68% of their population having received therapeutic hypothermia. Both of the case studies were published more than 5 years ago with data collected in excess of 15 years ago during which time the standard of care for NE is likely to have been different from the current practices. All three studies reported on sensitivity, specificity, PPV, and NPV.

A variety of standardized tools were used for CP diagnosis between the three studies. Additional file 4: Table 7 details the key findings and the outcomes evaluated, with the limitations identified by the authors. Glass et al. [83] reported on the absence of FM for the prediction of CP. Their findings were higher for the specificity 96–98% than sensitivity 29–50% for any CP and for moderate to severe CP, respectively. Notably, their NPV was 90% for any CP and 98% for moderate to severe CP, indicating that the presence of FM at 3 months is a strong indicator of an infant at low risk for a later diagnosis of CP, especially in the moderate to severe category. Although not part of the specific aims of our study, in the study by Glass et al. [83], it was significant that when they combined the Prechtl GMA and MRI findings, the NPV increased to 100%. In the study by Ferrari et al. [82] they reported that the presence of any CS movements between term and 4 to 5 months post-term had a sensitivity of 100% and a specificity of 68.7%, with a PPV 100% and a NPV of 78.3% for predicting CP. In the oldest study by Prechtl et al^.^ [84], the predictive ability in terms of the timing of the GMA was determined, that is, if done early, in the first 2 weeks of life versus late assessments between 15 and 22 weeks of life. Their findings were: sensitivity 100% and specificity 46.2%, with PPV 65.0% and NPV 100% for the early assessments, compared to late assessments with 84.6% across the board for sensitivity, specificity, PPV, and NPV. Neither of the case studies included infants receiving therapeutic hypothermia for NE which was not yet the standard of care. Ferrari et al. [82] identified selection bias as a limitation, where mild HIE as a contributor to NE may have been underrepresented due to these infants not being referred for evaluation. Prechtl et al. [84] did not state their limitations.

### Risk of bias

Even though this was a scoping review and did not require the critical appraisal of the three included articles, the critical appraisal tool for JBI [85, 86] helped to assess the quality of the articles and identify the differences and similarities between these two case studies. These main points are summarized here, and details are presented in Additional file 5: Table 8.

The quality of evidence derived from a review is largely dependent on the quality of the studies included.

This observational prospective cohort study by Glass et al. scored 100% in 10 of 11 questions [83]. This therefore assesses this study to be of high quality. The single question for which the study did not score 100.0% was that of the strategies used to address incomplete follow-up. Incomplete follow-up may result in selection bias. According to the JBI method, it is important that all the outcomes are assessed and participants with unequal follow-up periods must be taken into account in the analysis. For this study, patients with incomplete follow-up were not analyzed. If the analysis was not statistically feasible, this was not stated in the study.

Neither of the case studies scored 100% on all ten questions. The two case studies scored 100% for six of the ten questions on the checklist. These questions assess the two included case studies as being moderate-quality case series as there were limitations. They had good scores for using valid methods for the identification of the condition for all participants, having clear reporting of the demographics of the participants in the study, as well as, having clear clinical information of the participants. The outcomes of the cases were clearly reported for both studies. They also had clear reporting of the presenting site demographic information and used appropriate statistical analysis.

According to the JBI method, for the study participants, the authors should provide clear exclusion criteria. These inclusion and exclusion criteria should be specified with sufficient details and all the necessary information critical to the study. While Ferrari et al. [82] did fulfill these criteria, of note, Prechtl et al. [84] did not state their exclusion criteria, so this may limit the generalizability of the results. For good-quality case series, the study should clearly describe the method of measurement of the condition. This should be done in a standard (i.e., same way for all patients) and reliable (i.e., repeatable and reproducible results) way. The clinical condition for our study is NE. Both case studies listed a number of criteria for possible inclusion for NE but did not state the number or combination of these criteria required for the diagnosis and so scored 0.0% for this question. They did use a standard, albeit different, method for NE severity, with Ferrari et al. [82] used the Sarnat staging [7] while Prechtl et al. [84] used the Levene method [87]. With regard to the consecutive inclusion, studies that indicate a consecutive inclusion are more reliable than those that do not. Neither of our included studies stated clearly if they did consecutive inclusion of every neonate meeting the inclusion criteria, at their institutions, during the identified periods. Thus, they both scored 0.0% for this. Along a similar vein, the completeness of a case series contributes to its reliability. Studies that indicate a complete inclusion are more reliable than those that do not. Neither Ferrari et al. [82] nor Prechtl et al. [84] clearly stated that they included all the patients in their studies and scored 0.0% for this question.

The biases include selection, information, and sampling variation. Selection bias is typical of case series as it is a choice of a series of patients with a particular illness (NE), and a suspected linked outcome (CP) [88]. Selection bias limits the generalizability of the results. Information bias is less in retrospectively collected data as it is determined by what is already documented in the medical chart. These three studies were prospectively collected data making them susceptible to information bias. With regard to sampling variation, the precise determination of the rate of a disease, other than by chance, requires a large sample size. All of the included studies can be described as employing small sample sizes, and Glass et al. [83] had the highest number of participants at 58, while Ferrari et al. [82] had 34 cases and Prechtl et al. [84] had 26 cases with a follow-up period of over 3 to 4 years. Sample size may have been limited by the collection method as no study stated if they were inclusive of every neonate meeting the inclusion criteria, at their institutions, during the identified periods.

## Discussion

The scoping review methodology provided valuable insight into the current limited state of knowledge on the use of the GMA in the term neonate diagnosed with NE to predict CP by the age of 2–3 years. In fact, in the late preterm infant with NE, there was no evidence that met our inclusion criteria. Furthermore, the current evidence for our population was until recently, with the study by Glass et al. [83] derived from two case studies which do not constitute a level of evidence on which we can base definite recommendations. Our scoping review results highlight the need for more specific, higher-quality research in this area.

From the review, we were able to glean some important insights into the use of the GMA in our population. In the study by Glass et al. [83], infants with absent FMs at 3 months should be monitored closely as they are at high risk for a diagnosis of moderate to severe CP. The high NPV indicate that if FMs are present at the 3-month assessment of the GMA, the outcome is unlikely to be moderate to severe CP. In addition, a normal neonatal MRI in combination with a normal GMA at 3 months in a term high-risk infant is likely associated with a low risk for moderate to severe CP. This is reassuring advice that can be given to parents. In both case studies, the presence of the CS movement pattern of the GMA does correlate with the prediction of CP by 2 years of age. Prechtl et al.[84] noted that NE has an effect on spontaneous movements in term neonates, be it transient or persistent. Early assessments may be unable to differentiate between abnormal spontaneous movements that may be transient from those that will persist and eventually be associated with CP. Early assessments do not give as good predictive values as later assessments; therefore, the trajectory of the GMA may be a more significant indicator of outcomes than a solitary assessment [46, 84].

### Limitations of the included studies

Our findings support the role of the GMA as a good tool for the prediction of CP for those infants born at term with NE. There are however limitations to consider including the following: two of the three publications were case series, the variability in the NE definitions, the date of the publications identified and neither study contained neonates treated with the now standard of care, therapeutic hypothermia. Another important limitation is the low number of studies meeting our inclusion criteria.

Firstly, internal validity is likely to be low, as occurs especially in case series, since there are no comparator groups exposed to a similar array of variables. External validity would similarly be limited. Since this scoping review only represents level IV evidence [89], it reveals the need for future research in this area since it suggests that neonates with NE, at least those at term, may benefit from follow-up assessments with the GMA to help earlier identification of CP.

Secondly, in terms of the definition of NE, for the study by Ferrari et al. [82], there were some differences in the way NE was defined. In general, the standard accepted criteria that define NE were used, but they stated that study participants had different combinations of the NE criteria. Evidence shows that the etiology [8, 16] of encephalopathy as well as its severity may influence the outcomes [8]. Although the severity of NE (that is, mild, moderate, or severe reflected by the Sarnat stage [7]) was assessed in the study, no further differentiation of severity as it related to the predictive ability for CP was done. This may have been due to the small sample size of the study (*n* = 34) and the inevitable decreased power that would have resulted from subdivisions. Prechtl et al. [84] also subdivided the NE diagnosis into mild to moderate (*n* = 13) and severe (*n* = 13) NE. Similar to Ferrari et al. [82], the outcomes were not reported according to these NE subdivisions.

Thirdly, in terms of the timing of the publications, both of the case studies were more than 5 years old. Management has changed over time, and updated data in this evolving area would be beneficial. It is however interesting that the older study by Prechtl et al. [84] in 1993 supports the same later findings of the Ferrari et al. [82] study in 2011 with respect to the predictive ability of the GMA. This lends support to the reliable role of the GMA in the identification of those children born at term with NE who are at risk for CP.

Fourthly, neither of the case studies seemed to have been done in neonates treated with therapeutic hypothermia, which is the current standard of care [3, 4]. We are therefore uncertain if therapeutic hypothermia changes the quality of the GM, and if it does change, how long might this persist. Information like this is important to inform the timing of the early GMA post-therapeutic hypothermia intervention. Similar consideration had to be done for identifying the optimal window for cranial MRI in neonates treated with therapeutic hypothermia [90]. This lends credence to the gap in research in this area of NE and its association with CP.

Finally, low sample sizes in all the studies limit the power of the studies.

### Strengths and limitations of this review

The strength of our review primarily lies in the scoping review methodology that we chose. This method was advantageous as it facilitated an exhaustive search of the literature to define the current extent of knowledge and so allowed the research gaps to be identified.

We appreciated that there were several limitations. Firstly, we excluded studies that were not in English. This may be significant as there were only three studies identified in the review that met the established inclusion criteria. Therefore, with this limited number, any additional studies may have impacted our results. Secondly, the ages of the studies are of concern. Only one study was published recently in 2021 [83] with participants recruited between the years 2015 to 2017 and included patients that received therapeutic hypothermia. The study that was published in 2011 by Ferrari et al. [82], recruited participants between 2003 and 2006. The cohort of the Prechtl study was recruited between 1985 and 1989. Medical management has evolved since these two case studies to include strategies such as therapeutic hypothermia [3, 4]. The impact of this on our results is unknown. Lastly, we recognize that using a cutoff of CP diagnosis by 2–3 years of age constitutes a limitation for a number of reasons. Mild motor impairments may resolve with early intervention and not be eventually classified as a CP diagnosis. On the other hand, even milder forms of CP may not be identified early by 2–3 years of age and become apparent when more higher function motor tasks are required at older ages.

### Suggestions for further research

This review elucidates multiple potential areas for research.

#### Quantitative research

Meta-analysis—a meta-analysis of the data was not possible due to the minimal number of studies available. As more data becomes available, more accurate suggestions, for the use of the GMA in our population, can be made.

Prospective study—to provide higher quality evidence, research would preferably be a multi-center prospective cohort study, with matched low-risk controls. Standardization to the Prechtl GMA and the same assessment for CP at the same postnatal age would also add to the quality of the study. A study looking at the use of the GMA and its detailed qualitative analysis, the Motor Optimality Score (MOS), in our population of term and late-preterm infants diagnosed with NE is needed. Medical management has changed since the era of the cohorts in two of the three studies included in this review. More studies with the link of the GMA with respect to NE severity, etiology, and contemporary management would be of benefit. Alkan et al. 2021 [91] published a case–control study looking retrospectively at the motor repertoire in term infants with HIE at 3–5 months post-term using the MOS. They found that the total MOS scores were lower than that of their neurotypical counterparts. They also found that the higher the severity of HIE, the lower the MOS score. This study provides up-to-date further support for the need for a large prospective cohort study in this area.

Predictive ability of GMA in CP severity in our population—determination of the predictive ability of the GMA, including the MOS, for the degree of functionality in CP, such as that determined by the Gross Motor Function Classification System (GMFCS) [74] scoring system would be beneficial for implementation of early intervention strategies.

Optimal timing of an early assessment—since the trajectory of the GM may provide significant clues to neurodevelopmental outcomes more research is needed in this area. Transient effects on the GM may result from medical management such as therapeutic hypothermia and medications including sedatives and anticonvulsants. The optimal timing of the first assessment in this population requires further elucidation.

#### Qualitative research

Parental counseling and anticipatory intervention—parental perspectives on the use of the GMA as a predictor of neurodevelopmental outcomes in our population would be desirable for future research. This would influence counseling by the medical teams with regard to reassurance or the need for early intervention and the extent of neurodevelopmental follow-up.

## Conclusion

In term infants with encephalopathy, the predictive ability of the GMA is not as reliable when performed early versus later (at 15–22 weeks of age). The finding of normal GMs at 3 months of age is reassuring that a high-risk neonate is unlikely to develop moderate to severe CP. The finding of CS GM is a predictor for the diagnosis of CP by 2 years of age in the term population with NE.

Additionally, there are no existing studies specific to the application of the GMA in late-preterm infants with NE.

The deficit of high-quality research limits the applicability, so the GMA should not be used in isolation when assessing these populations. The evidence for this is limited by few studies and a lack of high-quality research. The evidence is lacking for the utilization of the GMA in these populations treated with therapeutic hypothermia. Furthermore, of possibly greater potential applicability is the inclusion of the MOS as a predictor of CP [92, 93] in term and late preterm infants with NE when treated with therapeutic hypothermia.

## Supplementary Information

**Additional file 1.** Protocol for GMA and NE in CPR2.

**Additional file 2.** Proposal.

**Additional file 3.** Search strategies.

**Additional file 4.** Tables for excluded and included studies.

**Additional file 5.** Critical appraisal.

**Additional file 6.** Tables for data extraction.

## Data Availability

Data sharing is not applicable to this article as no datasets were generated or analyzed during the current study. Materials during the current study are available from the corresponding author on reasonable request.

## Abbreviations

BW: Birth weight
CI: Confidence interval
CP: Cerebral palsy
CS: Cramped synchronized
EEG: Electroencephalogram
ELBW: Extremely low birth weight
ERP: Event-related potential
FM: Fidgety movements
GA: Gestational age
GM: General movements
GMA: General Movements Assessment
GMFCS: Gross Motor Function Classification System
HIE: Hypoxic-ischemic encephalopathy
HINE: Hammersmith Infant Neurological Examination
JBI: Joanna Briggs Institute
LR: Likelihood ratio
LBW: Low birth weight
MOS: Motor Optimality Score
Movement-ABC: Movement Assessment Battery for Children
MRI: Magnetic resonance imaging
NAPI: Neurobehavioral Assessment of the Preterm Infant
NBW: Normal birth weight
NE: Neonatal encephalopathy
NPV: Negative predictive value
n.s.: Not stated
PCC: Participant, concept, context
PMA: Postmenstrual age
PR: Poor repertoire
PPV: Positive predictive value
PRISMA-ScR: Preferred Reporting Items for Systematic Reviews and Meta-analyses extension for scoping review
TIMP: Test of Infant Motor Performance
TINE: Touwen Infant Neurological Examination
SD: Standard deviation
US: Ultrasound
VLBW: Very low birth weight
