# Supplementary material for: The assessment of general movements in term and late-preterm infants diagnosed with neonatal encephalopathy, as a predictive tool of cerebral palsy by 2 years of age—a scoping review
Source: Syst Rev. 2021 Aug 12;10:226. doi: 10.1186/s13643-021-01765-8 (PMC8359053; doi:10.1186/s13643-021-01765-8)
Supplement: Supplementary file 1 — Additional file 1. Protocol for GMA and NE in CPR2. [file 13643_2021_1765_MOESM1_ESM.pdf]

Research Square Preprint: Please note that this article has not completed peer review.

The general movements assessment in term and late-preterm infants diagnosed with neonatal encephalopathy, as a predictive tool of cerebral palsy by two years of age: a scoping review protocol.

CURRENT STATUS: UNDER REVIEW

Systematic Reviews BMC

Judy Seesahai
Sunnybrook Health Sciences Centre

Maureen Luther
Sunnybrook Health Sciences Centre

Carmen Cindy Rhoden
Sunnybrook Health Sciences Centre

Paige Terrien Church
Sunnybrook Health Sciences Centre

Elizabeth Asztalos
Sunnybrook Health Sciences Centre

Rudaina Banihani
Sunnybrook Health Sciences Centre

rudaina.banihani@sunnybrook.ca*Corresponding Author*
ORCiD: https://orcid.org/0000-0002-3865-3045

DOI:

10.21203/rs.2.20965/v3

SUBJECT AREAS

Obstetrics & Gynecology

KEYWORDS

Neonatal encephalopathy; general movement assessment; Prechtl; hypoxia-ischemia encephalopathy; cerebral palsy, infants/neonates, term babies, preterm babies, motor development.

Abstract

Background Prediction of long-term neurodevelopmental outcomes remains an elusive goal for neonatology. Clinical and socioeconomic markers have not proven to be adequately reliable. The limitation in prognostication includes those term and late-preterm infants born with neonatal encephalopathy. The General Movements Assessment tool by Prechtl has demonstrated reliability for identifying infants at risk for neuromotor impairment. This tool is non-invasive and cost-effective. The purpose of this study is to identify the published literature on how this tool applies to the prediction of cerebral palsy in term and late-preterm infants diagnosed with neonatal encephalopathy and so detect the research gaps. Methods We will conduct a systematic scoping review for data on sensitivity, specificity, positive and negative predictive value and describe the strengths and limitations of the results. This review will consider studies that included infants more than or equal to 34+0 weeks gestational age, diagnosed with neonatal encephalopathy, with a General Movements Assessment done between birth to six months of life and an assessment for cerebral palsy by at least two years of age. Experimental and quasi-experimental study designs including randomized controlled trials, non-randomized controlled trials, before and after studies, interrupted time-series studies and systematic reviews will be considered. Case reports, case series, case control and cross-sectional studies will be included. Text, opinion papers and animal studies will not be considered for inclusion in this scoping review as this is a highly specific and medical topic. Studies in the English language only will be considered. Studies published from at least 1970 will be included as this is around the time when the General Movements Assessment was first introduced in neonatology as a potential predictor of neuromotor outcomes. We will search five databases (MEDLINE, Embase, PsychINFO, Scopus and CINAHL). Two reviewers will conduct all screening and data extraction independently. The articles will be categorized according key findings and a critical appraisal performed. Discussion The results of this review will guide future research to improve early identification and timely intervention in infants with neonatal encephalopathy at risk of neuromotor impairment.

Background

Prediction of long-term neurodevelopmental outcomes remains an elusive goal for neonatology. Clinical and socioeconomic outcome markers have not proven to be adequately reliable[1,2]. The limitation in prognostication includes those term and late-preterm infants born with neonatal encephalopathy (NE).

NE describes those infants born with an atypical neurological exam and is by definition heterogeneous in etiology[3]. The specific etiology may not be clear for months to years later but the presentation is characterized by central nervous system disruption[4] and is associated with an increased risk for long-term neurodevelopmental challenges including cerebral palsy (CP). Infants presenting with NE are managed now with therapeutic hypothermia as the standard of care; this is presumptive management, and is time sensitive should the etiology be hypoxia/ischemia (Hypoxic Ischemic Encephalopathy (HIE)), in term and late-preterm infants[4,5]. Therapeutic hypothermia reduces the likelihood of challenging outcomes by containing any potential ongoing neurological injury. It does not, however, completely eradicate the possibility of long-term neurodevelopmental disability[6].

For parents of infants affected by NE, the desire for accurate prognostication is of tantamount importance[7]. This information can guide decisions around early intervention and, in severe cases, withdrawal of care for those infants with severe involvement. For those infants that survive NE and are at increased risk for CP, recent international recommendations now call for early detection and intervention of CP in order to improve functional outcomes[1,8,9]. These recommendations are based on mounting evidence for better detection tools as well as the benefits of early intervention.

Historically, clinical and radiological predictors of neurological outcomes were used to classify the degree of NE. Severity scoring systems include the classical grading by Sarnat and Sarnat[10] in 1976, to the newer scores by Miller et al.[11] in 2004, with added parameters such as oral feeding difficulties and the presence of seizures. Radiologically, specific findings of diffusion restriction on magnetic resonance imaging (MRI) have been linked to later development of CP[4]. These predictors, however, were not sufficiently accurate[1,2] and the high costs of imaging as well as shortages in access further

restricts the utility. Neurological examinations have historically been limited in predictive value but recent emerging evidence with an observational tool, the General Movements Assessment (GMA) developed by Dr. Heinz Prechtl has demonstrated strong predictive value[12, 13].

The GMA is a non-invasive, cost-effective tool with demonstrated reliability for identifying infants at risk for neuromotor impairment[14]. General movements (GMs) are complex, highly variable, whole-body movements which emerge in the fetus and progress through an age-specific developmental trajectory, dissipating by the end of the first four to five months of life[13]. Developmental progression and variety, or lack thereof, are indicators of nervous system integrity and can reflect neurodevelopmental outcomes[15]. Cramped synchronized (CS) and absent fidgety movements are considered abnormal GMAs, demonstrating developmental stereotypy[13].

Several researchers have looked at the GMA from different aspects. A preliminary search of PROSPERO, MEDLINE, the Cochrane Database of Systematic Reviews and the Joanna Briggs Institute (JBI) Database of Systematic Reviews and Implementation Reports was conducted to assess this research. There were two current systematic reviews on GMA, one in 2018[16] and the other in 2017[8]. In addition, eight older reviews were identified: seven systematic reviews[13,17-22] and one literature review[23] done between 2001 to 2013. The search also revealed three pending reviews identified around the topic of the predictive value of GMA[24-26]. These pending reviews were all systematic reviews.

The key characteristics and main findings of the above reviews on GMA are presented in Table 1, Appendix I. In general, the latest systematic review, by Kwong et al. in 2018[16], compared assessments of GMA and found that the Prechtl method had the best prediction of CP. In the 2017 systematic review by Novak et al.[8], their group reviewed the evidence for the best tools for early, accurate diagnosis and intervention in infants at risk for CP. They considered all gestational ages (GA) and all diagnoses for infants that were high-risk. They recommended a combined approach for early CP diagnosis including history, neuroimaging, standardized neurological, and standardized motor

assessments, to facilitate timely diagnosis and intervention. The other systematic reviews and literature review were all more than five years ago with the latest in 2013[13]. The findings of these older reviews are also summarized in Table 1. Similar to the latest two reviews, the older reviews either looked at preterms or all GA groups and diagnoses.

Of the three pending systematic reviews identified in PROSPERO, the oldest review protocol (Kwong et al.)[26] was registered in 2016 by similar authors of the 2018 review mentioned above. The next review protocol was registered in February 2018 by Raghuram et al.[24], and plans restrictions to preterms with all diagnoses, specifically examining automated movement recognition technology with the GMA. The third review protocol, registered in April 2018, by Angélica Valencia[25] is limited to preterm infants and is evaluating the type of method used for the recognition of the GMA, not the relationship of the GMA to neuromotor outcomes. None of these reviews specifically look at the population we identified for this scoping review, that is, term and late-preterm infants with NE. Thus, a gap exists in the literature to clearly identify the evidence for this specific population.

The objective of this review is therefore, to identify the scope of the research with regards to the GMA and its ability to predict CP, in term and late-preterm infants with a diagnosis of NE, and to identify the gaps in the literature.

Table 1

Summary of reviews (published and pending) on the general movements assessment and its predictive value for neuromotor outcomes

Article	Date of publication	Country	Type of studies included	Population (general characteristics)	GM assessment	Key findings	Predictive value
Published systematic reviews:							
Kwong et al.[16]	2018	Australia	Cohort studies	All GA with a GM. GM assessment done between 37 wks to < 5 months of age	Prechtl, Hadders-Algra and Hammersmith	47 studies<br>Fidgety movements using Prechtl method had the best prediction of cerebral palsy	Sensitivity 97% (95% CI 93-99)<br>Specificity 89% (95% CI 83-97) but had false positive results so cannot be used in isolation
Novak et al.[8]	2017	Australia	Systematic reviews and evidence-based clinical guidelines	All GA<br>All high-risk or low birth weight	Prechtl and Hammersmith	6 systematic reviews and 2 evidence-based clinical guidelines.<br>For infants with newborn-detectable risks before 5 months' corrected age, the GMs assessment plus neonatal MRI is > 95% accurate.<br>For infants with infant detectable risks after 5 months' corrected age, the HINE plus neonatal MRI is more than 90% accurate and is therefore recommended	Sensitivity 98% for Prechtl GM assessment
Bosquanet et al.[13]	2013	Australia	English studies only of randomized control trials and cohort trials both prospective and retrospective	23-41 weeks GA<br>All GA in high-risk populations<br>1358 participants	Any GM assessment type	19 studies<br>GM assessment had the best evidence and strength for prediction of cerebral palsy in < 5-year olds compared to neurological examination, MRI and cranial ultrasound. 1 of the 19 studies was term babies, rest preterm	Overall-<br>Sensitivity 98% (95% CI 74–100%)<br>Specificity 91% (95% CI 83–93%)<br>respectively:<br>- cranial ultrasound 74% (95% CI 63–83%) and 92% (95% CI 81–96%) respectively<br>- neurological examination 88% (95% CI 55–97%) and 87% (95% CI 57–97%) respectively.<br>MRI performed at term corrected age (in preterm infants) is a strong predictor of CP, with sensitivity ranging in individual studies from 86 to 100% and specificity ranging from 89 to 97%

Table 1 continued

Article	Date of publication	Country	Type of studies included	Population (general characteristics)	GM assessment	Key findings	Predictive value
Noble et al.[17]	2012	Australia	Not stated, possibly all types	All preterm infants	Any GM assessment tool	Reviewed clinimetric properties of longitudinal neonatal neurobehavioural and neuromotor assessments showed GMs was best for prediction of future outcome while the TIMP has best evaluative validity	Predictive validity for CP at 12 to 24 months was high for GMs:<br>- Sensitivity 100%<br>Interrater reliability:<br>- TIMP intraclass correlation=0.95 (strong)<br>- GMs (K=0.8)<br>- NAPI r=0.67–0.97 (moderate)
Zuk[18]	2011	Israel	Longitudinal follow up studies mainly	All GA<br>Heterogenous diagnoses	Prechtl	37 studies<br>35 longitudinal follow-ups, 2 n.s.	CS GMs that are consistent are highly predictive for CP.<br>FMs were a sensitive predictor of neurodevelopmental outcome in different populations of infants.<br>Sensitivity > 90% for these FMs and neurodevelopmental outcomes.<br>Sensitivity > 90% for a normal outcome when the GMs were normal
Darsaklis et al.[19]	2011	Canada	Longitudinal cohort studies in English and French	All GA	Pretchl's method only	39 studies	Conflicting evidence on whether the quality of fidgety or writhing movements is more accurate in predicting neurodevelopmental outcomes at 4 points in follow up – 12 to 23 months, 2 to 3 years, 4 to 11 years and 12 to 18 years
Burger et al.[20]	2009	United Kingdom	Descriptive research studies with predictive value at 12- or 24-months corrected age	All GA<br>1926 participants	Pretchl's method only	17 studies<br>Qualitative assessment of GM especially in the fidgety stage may be prognostic of neurodevelopmental impairment but the validity of the included studies was uncertain and o GM assessments should be used in combination with neuro-imaging or standardized neurological evaluations	Average score of the studies<br>- 8.82 (73.5%) from a total of 12 (SD 0.73).<br>- 15 of 17 studies showed high relationship (sensitivity 92%; specificity 82%; $p < 0.01$) in fidgety movements' period

Table 1 continued

Article	Date of publication	Country	Type of studies included	Population (general characteristics)	GM assessment	Key findings	Predictive value
Spittle et al.[21]	2008	Australia	Only studies in English	Clinimetric tools to predict outcomes in preterm infants (less than 37 week's GA) during their first year of life	Any GM assessment	The AIMS, TIMP, and GMs demonstrated the highest levels of overall reliability. Selection of motor assessment tools during the first year of life for infants born preterm will depend on the intended purpose of their use for discrimination, prediction, and/or evaluation	Overall reliability:<br>- 18 different assessment types<br>- the AIMS, TIMP, and GMs had the highest levels of (interrater and intrarater intraclass correlation coefficient or κ>0.85)
Hadders-Algra et al.[22]	2001	The Netherlands	Research reports	Preterm and term	Any GM assessment	Absence of complexity and variety of GM at 2-4 months post-term have a high risk for CP	Nil
Published literature review:							
Santos et al.[23]	2008	Brazil	Any study with evaluation tests to detect developmental disorders	All GA<br>All high-risk diagnoses	Prechtl	174 studies	GM reliability rate:<br>- Sensitivity: 100%<br>- Specificity: 96%
Proposed reviews from PROSPERO (published protocols):							
Raghuram et al.[24]	Feb 2018	Canada	Any study, including randomized, quasi-randomized, non-randomized clinical trials, retrospective or prospective observational trials, pilot studies, and feasibility studies	Preterm and term neonates with all high-risk diagnoses and the ability of an automated movement recognition technology for GM Assessment to predict motor impairment	Details not provided	Pending review completion	
Table 1 Continued							
Valencia[25]	Apr 2018	France	Any study in English, French or Spanish	Preterm before the term age with any diagnostic.<br>Evaluating the evidence on discriminative and predictive validity of the GM assessment performed on preterm infants on neurodevelopmental outcome	Details not provided	Pending review completion	
Kwong et al.[26]	July 2016	Australia	Cohort predictive studies, prospective or retrospective	> 37 weeks GA<br>Infants assessed between more than or equal to 37 weeks gestational age - less than 5 months corrected age and reassessed at any age for CP	Details not provided	Pending review completion	

Note. AIMS = The Alberta infant motor scale, CI = confidence interval, CP = cerebral palsy, CS = cramped synchronized, GM = general movements, HINE = Hammersmith Infant Neurological Examination, NAPI = Neurobehavioural Assessment of the Preterm Infant, n.s. = not stated, TIMP = Test of infant motor performance, wks = weeks

Methods/design

Review Question

The primary research question for this review is: What is the published data on the predictive value of

the GMA for the diagnosis of CP by two years of age in infants born at term or late-preterm presenting with NE?

The secondary research question is: What is the gap in the literature when the GMA is used to predict CP by two years of age in infants born at term or late-preterm presenting with NE?

Study Design

A scoping method is chosen for this type of review as to fulfilling of the objective of the review it requires searching and assessing a wide range of research methodologies involving the use of the GMA in CP prediction. A scoping review will capture all types of relevant research on the topic in a systematic, transparent, rigorous and reproducible manner. This scoping review will be conducted in accordance with the JBI methodology for scoping reviews[27]. The objectives, inclusion criteria and methods for this scoping review are detailed in advance and documented in a proposal (included as Additional file 1). The title of our review was registered with JBI.

Inherent in the nature of the scoping review is the inclusiveness of a wide range of literature, and so we anticipate differences in the data quality. Critical appraisal and data synthesis therefore will be challenging in terms of conclusive evidence as opposed to in a systematic review. The scoping review methodology is however especially advantageous to our question as these types of reviews target areas that have not been comprehensively assessed before.

Eligibility Criteria

The participant, concept, context (PCC) framework for scoping reviews will be used to define the review focus and can be found in Table 2.

Table 2

Inclusion and exclusion criteria for the prediction of CP by the GMA in late-preterm and term infants with NE

	Inclusion criteria	Exclusion criteria
Participants	Infants ≥ 34+0 weeks GA<br>Diagnosis of NE<br>GMA done between birth up to six months of life<br>Assessment for CP by at least two years of age	Infants born with:<br>life threatening congenital ab[illegible]<br>congenital viral infections<br>an abnormal karyotype and<br>metabolic disorders
Concept	GMA as a predictor of CP by two years of age is the main concept.	
Context	Studies that reported on:<br>- Infants with NE managed in hospitals and diagnosed by the standard of care (neurological history and examination)<br>- Studies from all countries that have outcomes reported in the acute neonatal and in the follow-up period by two years of age<br>- Studies in the English language only	

Note. CP = cerebral palsy, GA = gestational age, GMA = general movements assessment, NE = neonatal encephalopathy

Participants

This review will consider studies that include infants ≥ 34+0 weeks GA diagnosed with NE with a GMA done between birth to six months of life and an assessment for CP by at least two years of age (Table 2, Appendix II).

Reviews with infants born with life threatening congenital abnormalities, congenital viral infections, an abnormal karyotype and metabolic disorders will be excluded. Those studies without a GMA or with any automated application of the GMA will also be excluded.

Concept

GMA as a predictor of CP by two years of age is the main concept. Studies that report on sensitivity, specificity, positive predictive value (PPV) and negative predictive value (NPV) will be considered for inclusion. Detailed definition of concepts can be found in Table 3.

Context

This review will consider studies that reported on infants with an existing diagnosis of NE managed in hospitals and diagnosed by the standard of care assessment of a neurological history and examination. Studies will be considered from all countries that have outcomes reported in the acute neonatal and in the follow-up period by two years of age. Studies in the English language only will be considered as there is no team member with adequate language skills to translate from any other language.

Table 3
Definitions of concepts

Concepts	Definition
Neonatal encephalopathy	A clinically defined syndrome of disturbed neurologi the earliest days of life in an infant born at or beyon of gestation, manifested by a subnormal level of co or seizures, and often accompanied by difficulty wit and maintaining respiration and depression of tone reflexes[3]
Late-preterm	Neonates ≥ 34+0 to 36+6 weeks GA[28]
Term	Neonates 37+0 to 42+6 weeks GA[28]
Cerebral palsy	A group of permanent disorders of the development movement and posture causing activity limitations t attributed to non-progressive disturbances that occu developing fetal or infant brain[29]
General movements	These are spontaneous movements present from ea until about six months of life. GMs are variable, com movements that occur frequently, lasting long enou observed. The whole body is involved in a variable s limbs, neck, and trunk movements. Waxing and wan intensity, force and speed, they have a gradual begi end. They involve rotations along the limb axis. Slig direction are responsible for their fluid elegance. Im the nervous system cause the loss of GMs complexit variability resulting in monotonous and poor-quality Specific abnormal GM patterns have been identified predict later cerebral palsy:<br>1) Cramped-synchronized GMs – a persistence of rig movements that lack the normal fluidity. Contractio relaxations occur almost concurrently in limb and tr muscles.<br>2) The absence of fidgety GMs - fidgety movements movements of moderate speed with variable accele neck, trunk, and limbs in all directions. Normally, the predominant movement pattern in an awake infant at 3 to 5 months[30]
General movements assessment	A comfortably dressed infant, preferably with bare a legs, is videoed in supine position. The duration of t recording will depend on the age of the infant with p

	infants requiring up to 30 to 60 minutes. Term age a require 5 to 10 minutes of optimal recording. This re not require the observer’s presence. The trained obs reviews the recording later. The assessment is basec visual Gestalt perception without acoustic signal to r distraction. Two to three recordings of the preterm, c recording at term or early post-term age or both, anc one recording between 9- and 15-weeks’ post-term f basis of a developmental trajectory. An individual de trajectory indicates the consistency or inconsistency abnormal findings[30]
Sensitivity	The proportion of true positives that are correctly ide sample, or the true positive rate[31]
Specificity	The proportion of true negatives that are correctly ic sample, or the true negative rate[31]
Positive predictive value	The proportion of patients with positive test results v correctly diagnosed[32]
Negative predictive value	The proportion of patients with negative test results correctly diagnosed[32]

Note. GA = gestational age, GMs = general movements

Search strategy

A range of electronic databases will be searched to include medicine, nursing, allied health professions, sociology, psychology, education and social work. This scoping review will consider both experimental and quasi-experimental study designs including randomized controlled trials, non-randomized controlled trials, before and after studies and interrupted time-series studies. Case reports, case series, case control and cross-sectional studies will be included. In addition, systematic reviews that meet the inclusion criteria will be considered. Text and opinion papers will not be considered for inclusion in this scoping review as this is a highly specific and medical topic. Animal studies will not be included. Studies published from at least 1970 will be included as this is around the time when the GMA was first introduced in neonatology as a potential predictor of neuromotor outcomes[12]. The reference lists of articles will be scanned and experts in the infant developmental field will be consulted to identify studies relevant to our topic.

The search strategy will be phased, firstly created in Ovid Medline using a combination of index terms

and keywords around general movements, Prechtl, brain disease, HIE and perinatal asphyxia. An initial limited search of Ovid Medline, Embase and PsychINFO was undertaken to identify articles on the topic (See Additional file 2). There were no previous similar reviews. The text words contained in the titles and abstracts of relevant articles, and the index terms used to describe the articles from this limited search will then be used to develop a more refined full search strategy in the second phase, for MEDLINE, Embase, PsychINFO, Scopus and CINAHL (Appendix III). The search strategy, including all identified keywords and index terms, will be adapted for each included information source.

Study selection

EndNote X9 will be used for citation collation. Duplicates will be removed manually. Covidence will be used for screening by two independent reviewers (JS and ML). Disagreements will be resolved through a third reviewer (RB). The results of the search will be reported in a Preferred Reporting Items for Systematic Reviews and Meta-analyses extension for scoping reviews (PRISMA-ScR) flow diagram[33].

Data extraction, analysis and synthesis

Publications meeting the inclusion criteria will have a full text review to validate their eligibility. Each article will be assessed independently by two authors (JS and RB). Extraction will be done after full text screening using a data extraction tool developed by the reviewers. Excluded studies closely meeting the inclusion criteria will be included in a separate table as they may contain many elements of our inclusion criteria but not present separately the specific criteria of our interest. Further investigation of their data may provide significant results. Authors will be contacted to access further information and reassess eligibility of these studies. Excluded studies will be documented with reasons for their exclusion.

The data extracted from the identified studies will include specific details about the population, concept and context. Two tables will be generated with the first table having information on the key characteristics of each study, including author, year of publication, geographical setting, type of study, demographics of the participants, period over which the study was conducted, the method of identification of neonates at high-risk, if therapeutic hypothermia was instituted as management for NE, type of spontaneous movement assessment used, age at which participants were assessed, the

age at which CP was diagnosed and the methods used for neurological examination in the studies. The second table will have information on the key findings, the predictive indices used for the GMA in relation to CP (sensitivity, specificity, PPV and NPV), limitations of the studies and where relevant, reasons for exclusion in the studies that met most but not all of the inclusion criteria. These lists will be iterative. As the process evolves, the data extraction form may require modification to ensure all relevant information is included. Additionally, even though this was a scoping review and does not require a critical appraisal, the critical appraisal tool for JBI[34] will helped to identify differences and similarities between the included studies. The answers to the JBI critical appraisal tool will be detailed in a table.

Discussion

The extracted data will be presented in diagrammatic or tabular form in a manner that aligns with the objective of this scoping review. A narrative summary will accompany the tabulated and/or charted results and will describe how the results relate to the reviews objective and question. The critical appraisal result will also be tabulated and this will be used to further identify the strengths and limitations of the studies as well as the key findings in relationship to the objective of this scoping review. The strengths and limitations of our scoping review method on the credibility of the results will be detailed. The discussion and conclusions will reflect on the implications for future research and patient management.

Protocol amendments

Important amendments to the protocol will be reported with the results of the review.

What this study will add

This study will examine the scope of the literature with respect to the use of the GMA in NE for the prediction of CP. Assessment of the extent of the knowledge on this topic seems to have not previously been done. By inclusion of a critical appraisal of the available relevant literature, it will facilitate an appreciation of the quality of the existing knowledge in this area. It will therefore identify gaps in the research especially in the setting of NE management with therapeutic hypothermia.

List Of Abbreviations

CP cerebral palsy; CS, cramped synchronized; GMs, general movements; GA, gestational age; GMA, general movements assessment; HIE, hypoxic ischemic encephalopathy; JBI, Joanna Briggs Institute; MRI, magnetic resonance imaging; NE, Neonatal encephalopathy; NPV, negative predictive value; PCC, participant, concept, context; PPV, positive predictive value; PRISMA-ScR, Preferred Reporting Items for Systematic Reviews and Meta-analyses extension for scoping review.

Declarations

Ethics approval and consent to participate

Ethical approval will not be required as this is a scoping review of the literature and will not contain information directly identifying patients or content requiring patient consent.

Consent for publication

Not applicable

Availability of data and materials

Data sharing is not applicable to this article as no datasets were generated or analysed during the current study. Materials during the current study are available from the corresponding author on reasonable request.

Competing interests

The authors declare that they have no competing interests.

Funding

There is no funding required for this review.

Authors' contributions

First author: Judy Seesahai

Contributions: Substantial contributions to research design, acquisition, analysis and interpretation of data as well as drafting the paper.

Second author: Maureen Luther

Contributions: Contribution to acquisition, analysis and interpretation of data as well as well as involved in revisions to the paper.

Paige Terrien Church

Contributions: Substantial contributions to research design, analysis and interpretation of data as well as drafting the paper.

Carmen Cindy Rhoden

Contributions: Initial data search and drafting of paper.

Elizabeth Azstalos

Contributions: Substantial contributions to research design, acquisition, analysis and interpretation of data as well as drafting the paper.

Supervisor: Thomas Rotter

Contributions: Substantial contributions to research design and reviewing of the paper.

Principal Investigator: Rudaina Banihani

Contributions: Substantial contributions to research design, acquisition, analysis and interpretation of data as well as drafting the paper.

Acknowledgements

This review will contribute to a Master in Healthcare Quality degree for JS. The authors would also like to acknowledge the librarians that assisted with this research project, namely from the Sunnybrook R. Ian MacDonald Library, Henry Lam and Reena Besa, as well as the librarians Paola Durando and Sandra McKeown of the Bracken Health Sciences Library, Queen's University.

References

1. Finer NN, Robertson CM, Richards RT, Pinnell LE, Peters KL. Hypoxic-ischemic encephalopathy in term neonates: perinatal factors and outcome. J Pediatr. 1981 Jan; 98 (1):112-7. DOI: 10.1016/s0022-3476(81)80555-0
2. Campbell EE, Gilliland J, Dworatzek PDN, De Vrijer B, Penava D, Seabrook JA. Socioeconomic status and adverse birth outcomes: A population-based Canadian sample. Journal of Biosocial Science. Cambridge University Press; 2018;50(1):102–13.
3. (2014). Executive Summary: Neonatal Encephalopathy and Neurologic Outcome, Second Edition. Obstetrics & Gynecology, 123(4), 896–901. doi:

10.1097/01.AOG.0000445580.65983.d2.

4. Glass HC. Hypoxic-ischemic encephalopathy and other neonatal encephalopathies. [Review]. Continuum (Minneap Minn). 2018 Feb;57–71.

5. American Academy of Pediatrics, Committee on Fetus and Newborn. Hypothermia and neonatal encephalopathy. Pediatrics. 2014 Jun;133(6):1146–50.

6. Staub K, Baardsnes J, Hébert N, Hébert M, Newell S, Pearce R. Our child is not just a gestational age. A first-hand account of what parents want and need to know before premature birth. Acta Paediatr. 2014;103(10):1035–38.

7. Banihani R TCP. Neonatal Encephalopathy. In: Needelman H JB, editor. Follow-Up for NICU Graduates. 2018. p. 155–78.

8. Shepherd E, Salam RA, Middleton P, Han S, Makrides M, McIntyre S, et al. Neonatal interventions for preventing cerebral palsy: an overview of Cochrane Systematic Reviews. Cochrane Database of Systematic Reviews 2018, Issue 6. Art. No.: CD012409. DOI: 10.1002/14651858.CD012409.pub2.

9. Novak I, Morgan C, Adde L, et al. Early, accurate diagnosis and early intervention in cerebral palsy: advances in diagnosis and treatment. JAMA Pediatr. 2017;171(9):897–907. doi:10.1001/jamapediatrics.2017.1689.

10. Sarnat H, Sarnat M. Neonatal encephalopathy following fetal distress. Arch Neurol.1976.33:695-705.

11. Miller SP, Latal B, Clark H, Barnwell A, Glidden D, Barkovich AJ, et al. Clinical signs predict 30-month neurodevelopmental outcome after neonatal encephalopathy. American Journal of Obstetrics and Gynecology. 2004.190(1):93-99. DOI: https://doi.org/10.1016/S0002-9378(03)00908-6.

12. Einspieler C, Prechtl HFR, Bos AF, Ferrari F, Cioni G. In: Hart HM, Pountney M, Pearsall S (Editors). Developmental Medicine No. 167. Prechtl’s method on the

qualitative assessment of general movements in preterm, term and young infants. 1st ed. Mac Keith Press c2004. p ix - xi.

13. Bosanquet M, Copeland L, Ware R, Boyd R. A systematic review of tests to predict cerebral palsy in young children. Dev Med Child Neurol. 2013;55:418–26.

14. Heineman KR1, Hadders-Algra M. Evaluation of neuromotor function in infancy-A systematic review of available methods. J Dev Behav Pediatr. 2008: Aug 29(4):315-23. doi: 10.1097/DBP.0b013e318182a4ea.

15. Hadders-Algra M. General Movements: A Window for early identification of children at high risk for developmental disorders. J Pediatr. 2004 May 11;145(2 Supplement):S12–8.

16. Kwong AKL, Fitzgerald TL, Doyle LW, Cheong JL, Spittle AJ. Predictive validity of spontaneous early infant movement for later cerebral palsy: a systematic review. Dev Med Child Neurol. 2018 Feb 22;60(5):480-489.

17. Noble Y & Boyd R. Neonatal assessments for the preterm infant up to 4 months corrected age: a systematic review. Dev Med Child Neurol. 2012 Nov, 54: 129–39.

18. Zuk L. Fetal and infant spontaneous general movements as predictors of developmental disabilities. Dev Disabil Res Rev.2011;17:93–101. Available from: https://proxy.queensu.ca/login?url=http://ovidsp.ovid.com?T=JS&CSC=Y&NEWS=N&PAGE=fulltext&D=med7&AN=23362029 https://onlinelibrary.wiley.com/doi/abs/10.1002/ddrr.1104 https://dx.doi.org/10.1002/ddrr.1104

19. Darsaklis V, Snider LM, Majnemer A, Mazer B. Predictive validity of Prechtl’s method on the qualitative assessment of general movements: a systematic review of the evidence. Dev Med Child Neurol. 2011 Jun 17;53(10):896–906.

20. Burger M, Louw QA. The predictive validity of general movements – a systematic

review. Eur J Paediatr Neurol. 2009;13(5):408–20.

21. Spittle AJ, Doyle LW, Boyd RN. A systematic review of the clinimetric properties of neuromotor assessments for preterm infants during the first year of life. Dev Med Child Neurol. 2008 Apr 8;50(4):254–66.

22. Hadders-Algra M. Evaluation of motor function in young infants by means of the assessment of general movements: a review. Pediatr Phys Ther. 2001 Apr 1;13(1):27–36.

23. Santos RS., Araújo APQC., Porto MAS. Early diagnosis of abnormal development of preterm newborns: assessment instruments. J. Pediatr. (Rio J.) [Internet]. 2008 Aug [cited 2019 Aug 07];84(4):289-299. Available from: http://www.scielo.br.proxy.queensu.ca/scielo.php?script=sci_arttext&pid=S0021-75572008000400003&lng=en. http://dx.doi.org.proxy.queensu.ca/10.1590/S0021-75572008000400003.

24. Raghuram K, Orlandi S, Church P, Chau T, Uleryk E, Pechlivanoglou P, et al. Can an automated general movements assessment be used to predict motor impairment in high-risk infants? A systematic review and meta-analysis of diagnostic accuracy. PROSPERO International prospective register of systematic reviews. 2018 Apr 16; Available from: http://www.crd.york.ac.uk/PROSPERO/display_record.php?ID=CRD42018087892.

25. Valencia A. Discriminative and predictive validity of the general movements assessment: a systematic review. PROSPERO International prospective register of systematic reviews. 2018 Feb 14;Available from: http://www.crd.york.ac.uk/PROSPERO/display_record.php?ID=CRD42018088724.

26. Kwong AKL, Fitzgerald TL, Spittle AJ, Cheong JL, Doyle LW, Einspieler C. A systematic

review of the predictive validity of observational early infant motor assessments for subsequent cerebral palsy. PROSPERO International prospective register of systematic reviews. 2016; Available from: http://www.crd.york.ac.uk/PROSPERO/display_record.php?ID=CRD42016042551.

27. The Joanna Briggs Institute. The System for the Unified Management, Assessment and Review of Information (SUMARI) [Internet]. 2017 [cited 2019]. Available from: https://www.jbisumari.org/

28. World Health Organization. ICD-10 International Statistical Classification of Diseases and Related Health Problems: 10th Revision, Volume 2 Instruction Manual. www.who.int/classifications/icd/ICD-10_2nd_ed_volume2.pdf (Accessed on September 7, 2010)

29. Rosenbaum P, Paneth N, Leviton A, et al. A report: the definition and classification of cerebral palsy April 2006. Dev Med Child Neurol Suppl. 2007;109:8-14.

30. Einspieler C, Prechtl HFR. Prechtl's assessment of general movements: A diagnostic tool for the functional assessment of the young nervous system. Ment.Retard.Dev.Disabil.Res.Rev. 2005;11(1):61-6

31. Altman, D. G., & Bland, J. M. (1994). Diagnostic tests. 1: Sensitivity and specificity. BMJ (Clinical research ed.), 308(6943), 1552. doi:10.1136/bmj.308.6943.1552

32. Altman, D. G., & Bland, J. M. (1994). Diagnostic tests 2: Predictive values. BMJ (Clinical research ed.), 309(6947), 102. doi:10.1136/bmj.309.6947.102

33. Moher D, Liberati A, Tetzlaff J, Altman DG. Preferred Reporting Items for Systematic Reviews and Meta-Analyses: The PRISMA Statement. [Internet]. 2009 [cited 2019]. Available from: http://prisma-statement.org/PRISMAStatement/FlowDiagram.aspx

34. Moola S, Munn Z, Tufanaru C, Aromataris E, Sears K, Sfetcu R, Currie M, Qureshi R, Mattis P, Lisy K, Mu P-F. Chapter 7: Systematic reviews of etiology and risk. In:

Aromataris E, Munn Z (Editors). Joanna Briggs Institute Reviewer's Manual. The Joanna Briggs Institute, 2017. Available from https://reviewersmanual.joannabriggs.org/

Supplementary Files

This is a list of supplementary files associated with this preprint. Click to download.

PRISMA-ScR GMA and NE.docx
Additional file 2.docx
Additional file 1.docx
