# Supplementary material for: The assessment of general movements in term and late-preterm infants diagnosed with neonatal encephalopathy, as a predictive tool of cerebral palsy by 2 years of age—a scoping review
Source: Syst Rev. 2021 Aug 12;10:226. doi: 10.1186/s13643-021-01765-8 (PMC8359053; doi:10.1186/s13643-021-01765-8)

Additional file 3: Search strategies

Screening search


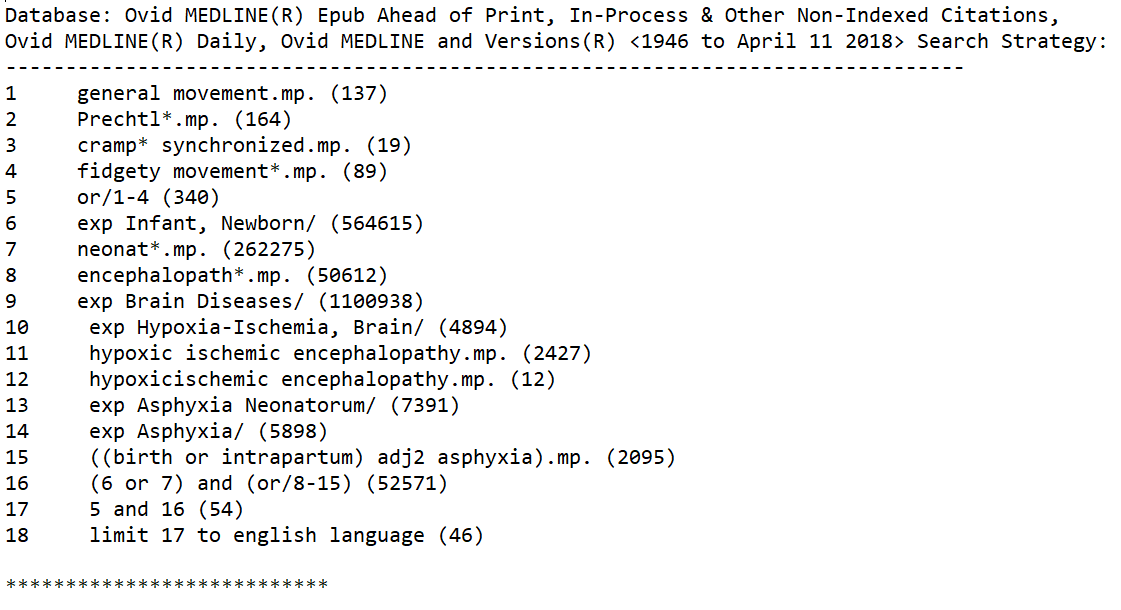


Dates of Ovid Medline screening search: conducted from 1946 to April 11^th^, 2018.


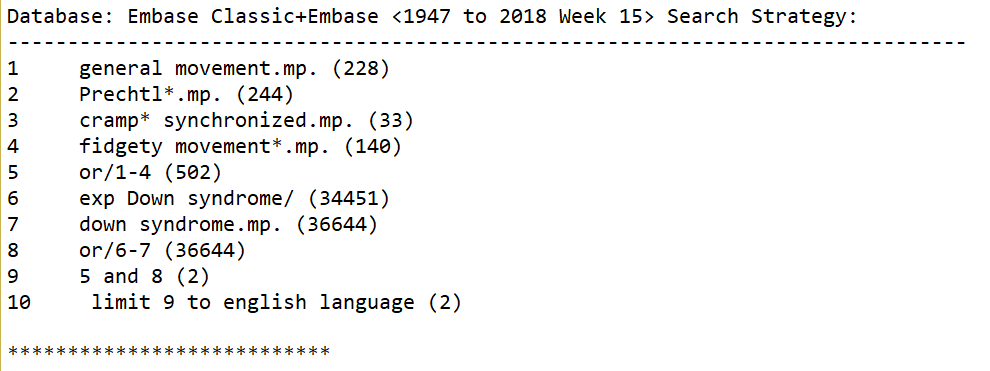


Dates of Embase screening search: conducted from 1947 to April 11^th^, 2018.


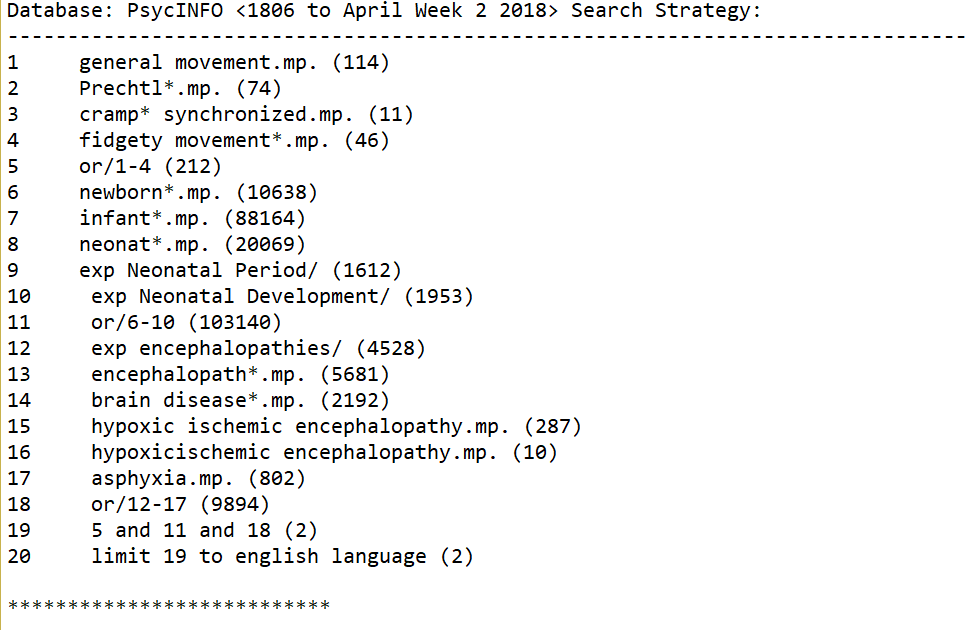


Dates of PsychINFO screening search: conducted from 1806 to April 11^th^, 2018.

Date of searches: First screening searches in Ovid Medline, Embase and PsychINFO, conducted on April 11^th^, 2018

Second search


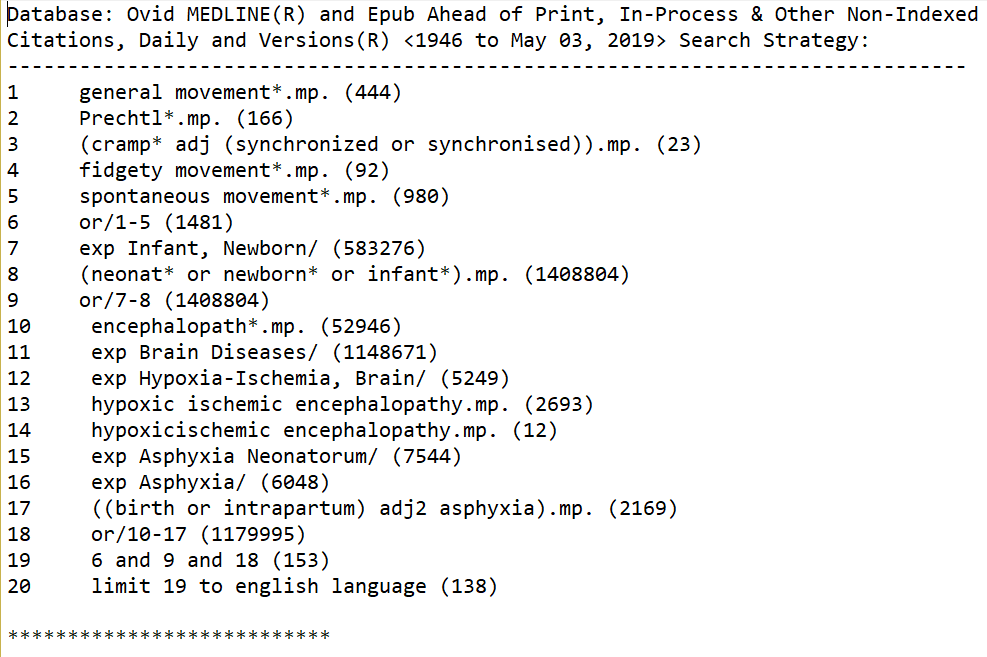


Dates of Ovid Medline second search conducted on May 3rd, 2019: for time period from 1946 to May 3^rd^ 2019.


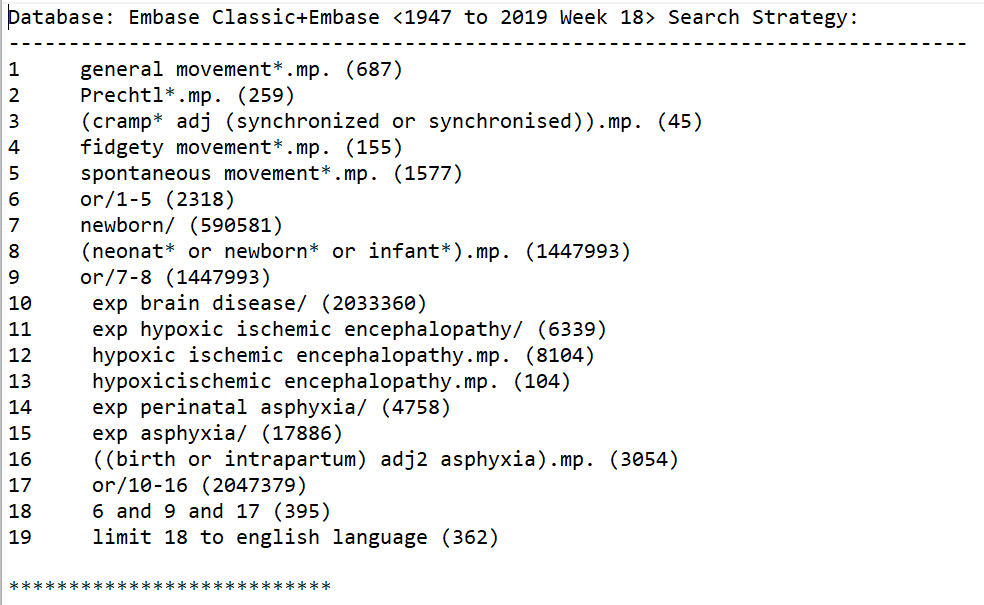


Dates of Embase second search conducted on May 3rd, 2019: for time period from 1946 to May 3^rd^ 2019.


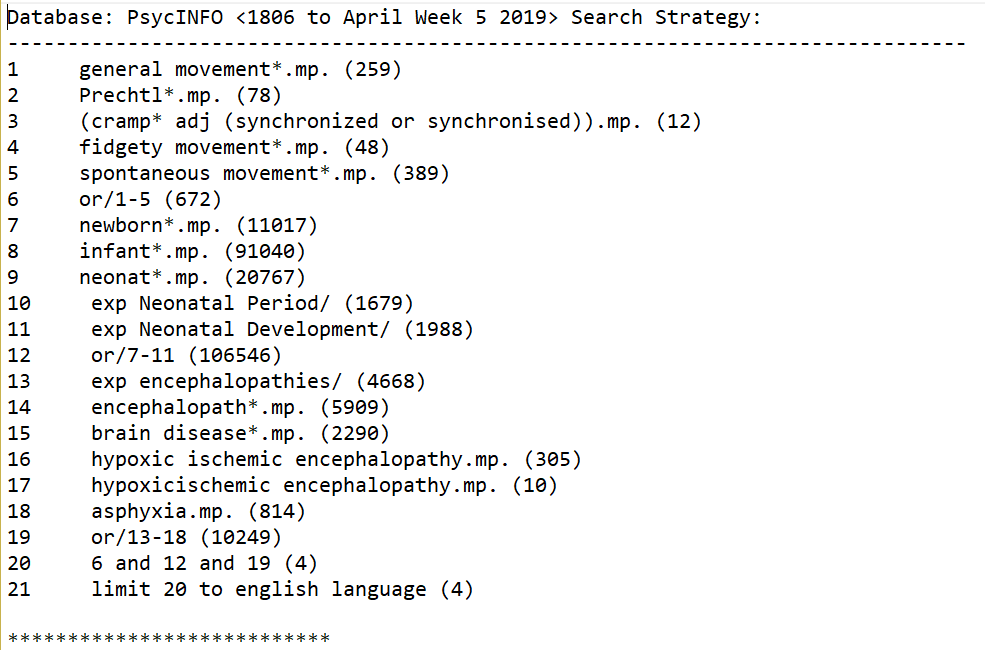


Dates of PsycINFO second search conducted on April 30^th^, 2019: for time period from 1806 to April 30^th^, 2019.


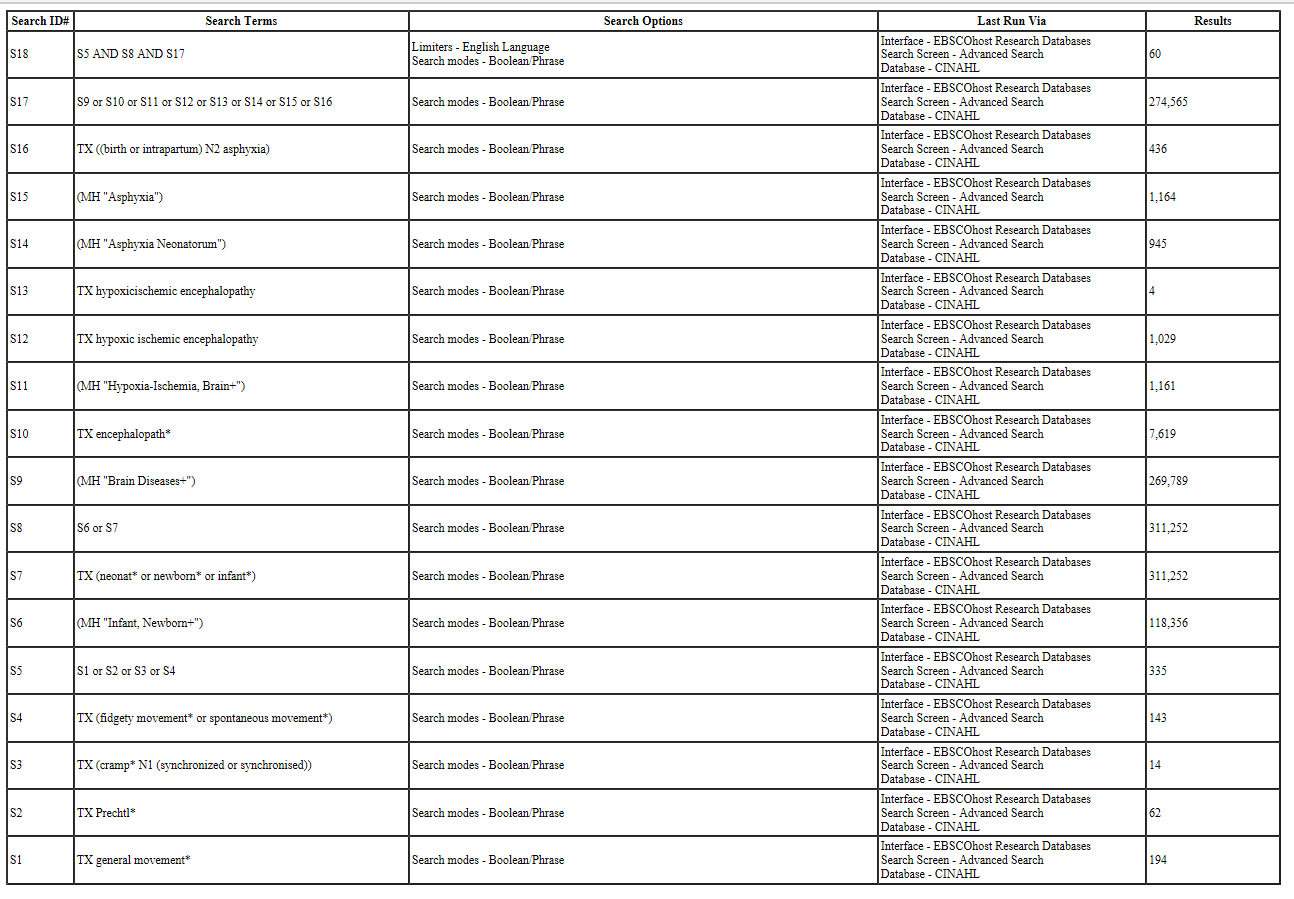


Dates of CINAHL second search conducted on May 11^th^, 2019: for time period from inception to May 11^th^, 2019.


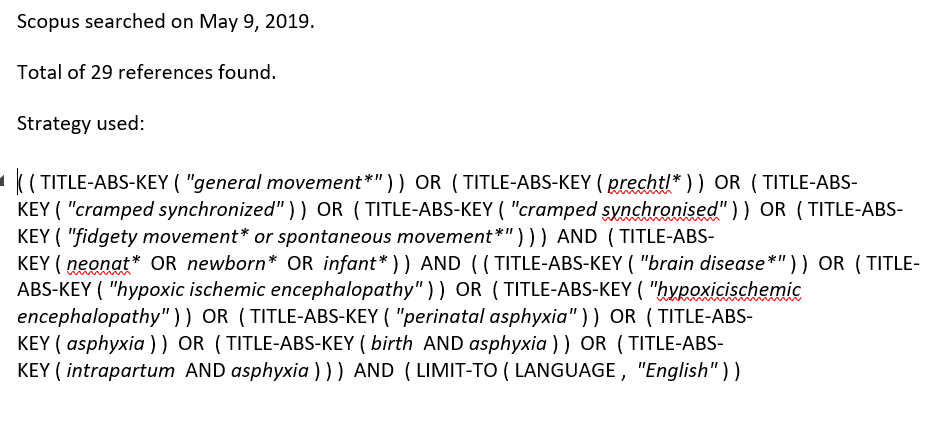


Dates of Scopus second search conducted on May 9^th^, 2019: for time period from 1946 to May 9^th^, 2019.

Third search

Search conducted on Ovid MEDLINE(R), Ovid MEDLINE(R) Daily and Epub Ahead of Print, In-Process & Other Non-Indexed Citations

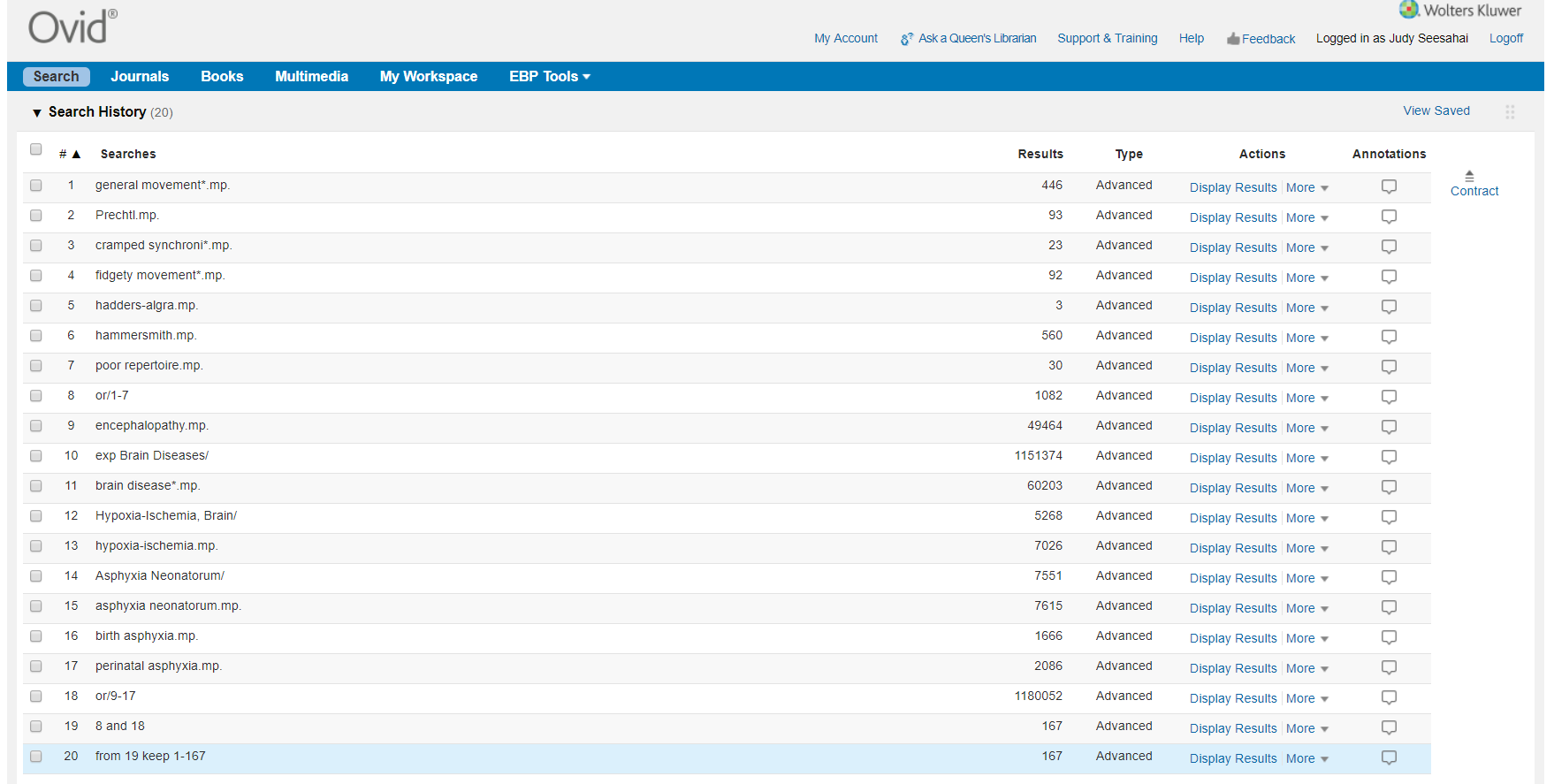


Dates of search: Third search conducted in Ovid Medline: from 1946 to May 9^th^, 2019.

Fourth and final search

Database: Ovid MEDLINE(R) and Epub Ahead of Print, In-Process & Other Non-Indexed Citations, Daily and Versions(R) <1946 to March 26, 2020>
Search Strategy:
--------------------------------------------------------------------------------
1     general movement*.mp. (476)
2     Prechtl*.mp. (170)
3     (cramp* adj (synchronized or synchronised)).mp. (24)
4     fidgety movement*.mp. (99)
5     spontaneous movement*.mp. (1025)
6     or/1-5 (1553)
7     exp Infant, Newborn/ (600543)
8     (neonat* or newborn* or infant*).mp. (1455661)
9     or/7-8 (1455661)
10     encephalopath*.mp. (55880)
11     exp Brain Diseases/ (1197704)
12     exp Hypoxia-Ischemia, Brain/ (5557)
13     hypoxic ischemic encephalopathy.mp. (2980)
14     hypoxicischemic encephalopathy.mp. (12)
15     exp Asphyxia Neonatorum/ (7650)
16     exp Asphyxia/ (6174)
17     ((birth or intrapartum) adj2 asphyxia).mp. (2265)
18     or/10-17 (1230874)
19     6 and 9 and 18 (159)
20     limit 19 to english language (144)

***************************​

Database: Embase Classic+Embase <1947 to 2020 Week 13>Search Strategy:
--------------------------------------------------------------------------------
1     general movement*.mp. (741)
2     Prechtl*.mp. (272)
3     (cramp* adj (synchronized or synchronised)).mp. (48)
4     fidgety movement*.mp. (167)
5     spontaneous movement*.mp. (1639)
6     or/1-5 (2432)
7     newborn/ (612117)
8     (neonat* or newborn* or infant*).mp. (1507899)
9     or/7-8 (1507899)
10     exp brain disease/ (2153053)
11     exp hypoxic ischemic encephalopathy/ (7167)
12     hypoxic ischemic encephalopathy.mp. (8991)
13     hypoxicischemic encephalopathy.mp. (105)
14     exp perinatal asphyxia/ (5041)
15     exp asphyxia/ (18545)
16     ((birth or intrapartum) adj2 asphyxia).mp. (3201)
17     or/10-16 (2167550)
18     6 and 9 and 17 (425)
19     limit 18 to english language (391)

***************************​

Database: APA PsycInfo <1806 to March Week 4 2020> Search Strategy:
--------------------------------------------------------------------------------
1     general movement*.mp. (268)
2     Prechtl*.mp. (78)
3     (cramp* adj (synchronized or synchronised)).mp. (14)
4     fidgety movement*.mp. (51)
5     spontaneous movement*.mp. (398)
6     or/1-5 (689)
7     newborn*.mp. (11352)
8     infant*.mp. (117547)
9     neonat*.mp. (21844)
10     exp Neonatal Period/ (1725)
11     exp Neonatal Development/ (2025)
12     or/7-11 (130975)
13     exp encephalopathies/ (4816)
14     encephalopath*.mp. (6144)
15     brain disease*.mp. (6718)
16     hypoxic ischemic encephalopathy.mp. (326)
17     hypoxicischemic encephalopathy.mp. (10)
18     asphyxia.mp. (1015)
19     or/13-18 (14573)
20     6 and 12 and 19 (9)
21     limit 20 to english language (9)

***************************​

Scopus Strategy. Searched on March 30, 2020. 31 references found.

( ( TITLE-ABS-KEY ( *"general movement*"* ) )  OR  ( TITLE-ABS-KEY ( *prechtl** ) )  OR  ( TITLE-ABS-KEY ( *"cramped synchronized"* ) )  OR  ( TITLE-ABS-KEY ( *"cramped synchronised"* ) )  OR  ( TITLE-ABS-KEY ( *"fidgety movement* or spontaneous movement*"* ) ) )  AND  ( TITLE-ABS-KEY ( *neonat**  OR  *newborn**  OR  *infant** ) )  AND  ( ( TITLE-ABS-KEY ( *"brain disease*"* ) )  OR  ( TITLE-ABS-KEY ( *"hypoxic ischemic encephalopathy"* ) )  OR  ( TITLE-ABS-KEY ( *"hypoxicischemic encephalopathy"* ) )  OR  ( TITLE-ABS-KEY ( *"perinatal asphyxia"* ) )  OR  ( TITLE-ABS-KEY ( *asphyxia* ) )  OR  ( TITLE-ABS-KEY ( *birth*  AND  *asphyxia* ) )  OR  ( TITLE-ABS-KEY ( *intrapartum*  AND  *asphyxia* ) ) )  AND  ( LIMIT-TO ( LANGUAGE ,  *"English”*

Dates of searches: Conducted on March 26-30^th^, 2020

Time period: Each database inception to March 30^th^, 2020.

**Additional search May 2021**

Date of search: Conducted on May 21^st^, 2021

Time period of search: January 1^st^ 2020 – May 21^st^, 2021

Ovid MEDLINE: Epub Ahead of Print, In-Process & Other Non-Indexed Citations, Ovid MEDLINE® Daily and Ovid MEDLINE® <1946-Present>

1 general movements.mp. 413

2 Prechtl*.mp. 189

3 cramp* synchronized.mp. 24

4 fidgety movements*.mp. 123

5 1 or 2 or 3 or 4 543

6 exp Infant, Newborn/ 624571

7 neonat*.mp. 306026

8 encephalopathy*.mp. 55844

9 exp Brain Diseases/ 1261639

10 hypoxic ischemic encephalopathy.mp. 3359

11 hypoxicischemic encephalopathy.mp. 13

12 exp Asphyxia Neonatorum/ 7794

13 exp Asphyxia/ 6330

14 ((birth or intrapartum) adj2 asphyxia).mp. 2400

15 6 or 7 765722

16 8 or 9 or 10 or 11 or 12 or 13 or 14 1294793

17 15 and 16 57858

18 5 and 17 112

19 17 and 18 112

20 limit 19 to (english language and yr="2020 -Current") 10

Screen shot of search


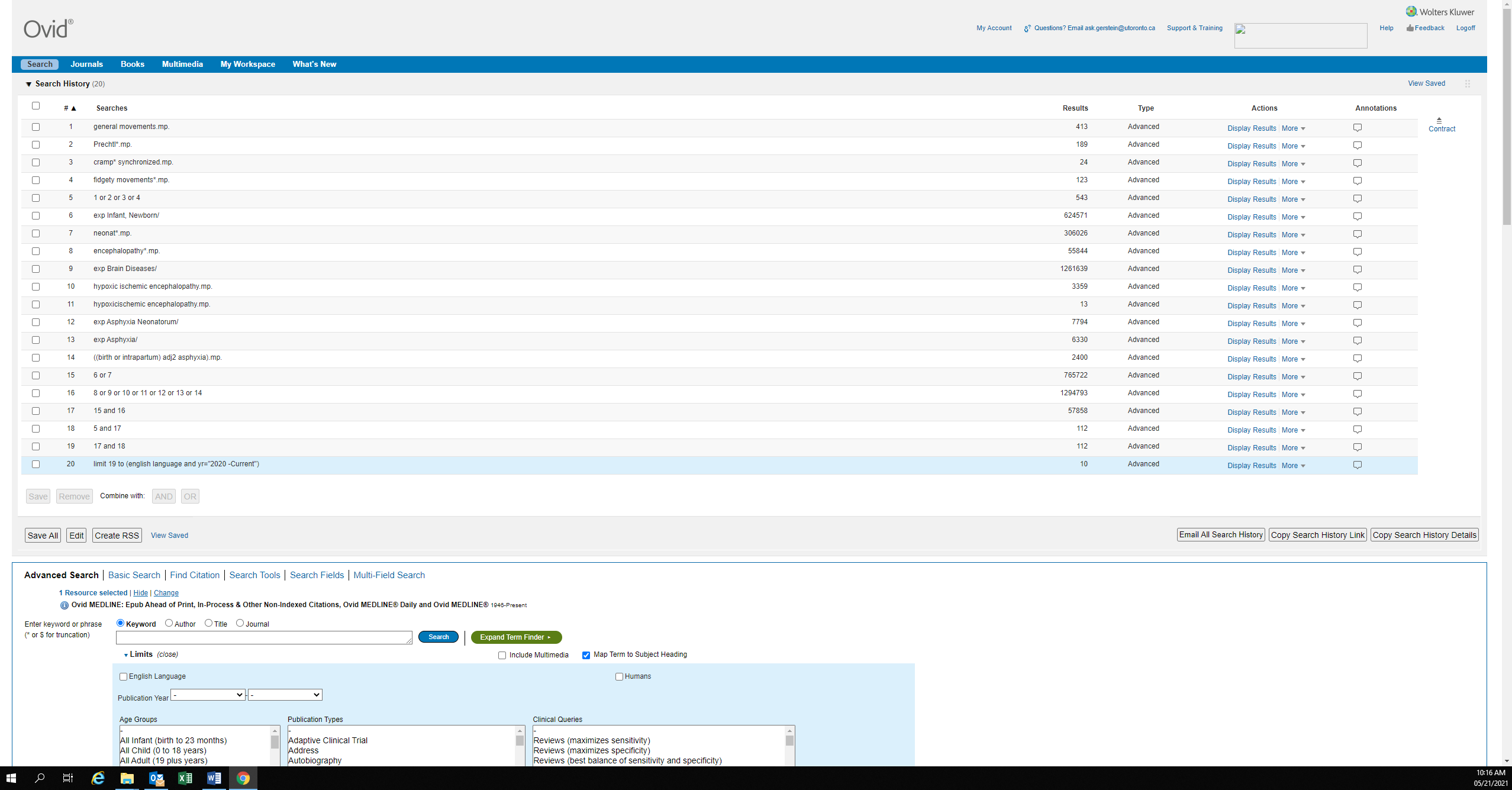

Supplement: Supplementary file 3 — Additional file 3. Search strategies. [file 13643_2021_1765_MOESM3_ESM.docx]
