# Supplementary material for: The assessment of general movements in term and late-preterm infants diagnosed with neonatal encephalopathy, as a predictive tool of cerebral palsy by 2 years of age—a scoping review
Source: Syst Rev. 2021 Aug 12;10:226. doi: 10.1186/s13643-021-01765-8 (PMC8359053; doi:10.1186/s13643-021-01765-8)
Supplement: Supplementary file 4 — Additional file 4. Tables for excluded and included studies. [file 13643_2021_1765_MOESM4_ESM.docx]

Additional file 4: Tables for excluded and included studies

| Table 4  *Key findings and characteristics of excluded studies for the GMA and the predictive ability for CP in late-preterm and term infants with NE* | | | | | | | | | | | |
| --- | --- | --- | --- | --- | --- | --- | --- | --- | --- | --- | --- |
| Article | **Date of publication** | **Country** | **Type of study** | **Population size (number of infants)** | **Population**  **(general characteristics)** | **Period of study** | **High risk identification** | **GMA** | **Age at GMA** | **Age of CP diagnosis** | **Method used for neurological examination** |
| Adde et al.(45) | 2007 | Norway | Prospective cohort | 74 high and low-risk | Gender: 33 males (44.6%), 41 females (55.4%)  GA:   - 42 (57%) preterm - 32 term (43%)   Preterm: 24-36 wks, (median 30.5 wks)  Term: >36 - <42 wks  Late preterm: n.s.  BW: 540-3800 g (median 1367 g)  High-risk: preterm 40%, term 25%.  NE: 5 (all term GA)  CP diagnosis: 3  Therapeutic hypothermia: 0 | n.s. | Presence of major US abnormalities  or MRI findings or the clinical history | Prechtl | 10-18 wks post-term | 9-31 months (median age 23 months) | Method not stated but information retrieved from medical specialist notes of their evaluation &  parental report |
| Brogna et al.(46) | 2013 | Italy | Cohort | 574 consecutive admissions | Gender: n.s.  GA: 34–36 completed wks  BW: 2299 ± 451  NE: n.s.  CP: 22 (4%)  Therapeutic hypothermia: 0 | Jan 2006- Dec 2010 | Admission to the Level II or III unit of the institution | Prechtl | 1-3 months post-term age | 24 months post-term age | Structured examination  in conformity with an extension of TINE 's criteria (70), and Bayley scale (71) |
| Cioni et al.(47) | 1997 | Italy | Case series | 58 term | Gender: 34 males (58.6%), 24 females (41.4%)  GA: 37-41 wks (mean 39 (SD 1))  BW: 1870 - 4350g (mean 3166 (SD 540)  NE: 38 (16 severe and 22 mild)  CP: 14  Therapeutic hypothermia: 0 | 1985 | Serial US scans | Prechtl | Every 3–4 wks from “first days of life” up to 65 wks | 13–31 months (median age 23 months) | Amiel-Tison and Grenier (69) examination, TINE 's criteria (70), Griffiths Scales (72) |
| Table 4 continued | | | | | | | | | | | |
| Article | **Date** | **Country** | **Type of study** | **Population size** | **Population**  **(general characteristics)** | **Period of study** | **High risk identification** | **GM A** | **Age at GMA** | **Age of CP diagnosis** | **Method used for neurological examinations** |
| Dekkers et al. (48) | 2020 | Netherlands | Prospective longitudinal cohort | 18 term | Gender: 12 males (66.7%) , 6 (33.3%) females  GA: 40.3 wks (1.4), mean (SD)  BW: 3423g (725), mean (SD)  NE: 18 (all HIE)  CP: 3 (27%)  Therapeutic hypothermia: 18 | 2009 - 2010 | History and examination, MRI | Hadders-Algra | 3 months | 2 qnd 5 years | Structured neurological examination, BSID-III (80), Movement –ABC, 2^nd^ edition (79) |
| Dimitrijević et al. (49) | 2016 | Serbia | Prospective cohort | 79 | Gender: 41 males (51.9%),  38 females (48.1%)  GA: 25-36 weeks  Late-preterm: ?33 or 36 (different number quoted in table versus text)  BW: definitive numbers for range n.s. but divided into categories ELBW (4), VLBW (16), LBW (58), NBW (1).  NE: 18 (late-preterm % n.s.)  CP: in late-preterm 1  Therapeutic hypothermia: 0 | Jul 2011- Dec 2013 | History and physical examinations | Prechtl | 1-3 months corrected age | 24 months corrected age | TINE 's criteria (70) and Surveillance of Cerebral  Palsy in Europe working group (SCPE) (73) |
| Einspieler et al. (50) | 2015 | China | Longitudinal study. Retrospective analysis of prospectively collected data | 61 | Gender: 46 males (75.4%), 15 females (24.6%)  GA:   - preterm: 29 (47.5%) - term: 32 (52.5%)   Late-preterm n.s.  BW: range n.s.  NE: 9 (GA n.s.)  CP: 10  Therapeutic hypothermia: 0 | Sep 2003 - Jun 2010 | Preterm birth or perinatal asphyxia at term, abnormal findings at pediatric examination or parental concerns | Prechtl | 9-16 weeks post term age | 2-3 years | n.s. but Classified by means of the GMFCS (74) at 3-5 years |
| Einspieler et al.(51) | 2019 | 24 sites worldwide (Europe, North America, South America, South Africa, Asia and Australia) | Retrospective | 468 | Gender: 58% male, 42% female  GA: (23- 42 weeks)   - preterm 56% - term 46%   Late preterm: n.s.  BW: 440-4500g  NE: yes, n.s. by GA  CP: yes, n.s. by GA  Therapeutic hypothermia: n.s. | 2012-2019 | Not detailed | Prechtl, including the MOS | 9-22 weeks post-term age | 2 years and 10 months - 5 years and 7 months | n.s. |
| Table 4 continued | | | | | | | | | | | |
| Article | **Date of publication** | **Country** | **Type of study** | **Population size** | **Population**  **(general characteristics)** | **Period of study** | **High risk identification** | **GMA** | **Age at GMA** | **Age of CP diagnosis** | **Method used for neurological examinations** |
| Feng et al. (52) | 2017 | China | Prospective case series | 110 high-risk | Gender: 71 males (64.5%), 39 females (35.5%)  GA:   - 28-37 wks: 65 - 37-40 wks: 35 - > 40 wks: 10   BW: n.s.  NE: 33  CP: 4  Therapeutic hypothermia: 0 | Jan 2012 - Jun 2013 | Unclear – possibly from clinical diagnosis | Prechtl | 1 month after delivery, done twice with a 1 wk interval | 1-year corrected age | Gesell developmental scale assessment (75) |
| Goyen et al. (53) | 2020 | Australia | Prospective longitudinal cross-sectional | 184 infants | Gender: n.s.  GA:   - preterm 101 (54.9%) - term 83 (45.1%)   BW: n.s.  NE: n.s.generally but reported as   - HIE 26 (14.1%) - neurological (25 (13.6%)   CP: 41 (22.3%) of which   - HIE 12   Therapeutic hypothermia: n.s. | 2011 - 2013 | Clinical diagnosis | Prechtl | 3 months | 2-3 years, 8.5 months (mean, SD = 4 months) | History, neurological examination and developmental motor assessment, Bayley scale (71) |
| Guzetta et al. (54) | 2007 | Italy | Prospective case series | 115 | Gender: 56 males (49%), 59 females (51%)  GA: 103 preterm and 12 term infants  Preterm: (wks), mean (SD)   - GA: 32.3 (2.8) range: 23–35 - late-preterm: n.s. - BW (g): mean 1776.3 (SD: 563.9), range: 500–3100 - CP: 4   Term: (wks): mean (SD)   - GA 39.6 (1.5), range: 37–41 - BW (g): mean 3471.1 (SD: 401.1) range: 2550–4100   - CP: 3  NE: n.s.  Therapeutic hypothermia: 0 | Jan 2002 - Apr 2003 | History of NE | Prechtl | 0-20 wks GA (used one closest to term age for the writhing period and to 12 weeks post-term age for the FM stage | At least at 18 months | Expanded version of the Amiel-Tison and Grenier (69) and  TINE 's criteria (70) |
| Table 4 continued | | | | | | | | | | | |
| Article | **Date of publication** | **Country** | **Type of study** | **Population size** | **Population**  **(general characteristics)** | **Period of study** | **High risk identification** | **GMA** | **Age at GMA** | **Age of CP diagnosis** | **Method used for neurological examinations** |
| Hadders-Algra et al. (55) | 1997 | Not stated | Case series | 16 | Gender: distribution n.s.  GA:   - 26-36 wks: 10 with 1 late-preterm - 38-43 wks: 6   BW:   - preterm: 850- 2750g - term: 2500- 3525g   NE: 7 (6 term and 1 late-preterm)  CP: 7  Therapeutic hypothermia: 0 | Not stated | History of HIE and an abnormal GMA | Prechtl | 1 wk - 4 months post-term | 18-31  months corrected age (median 19 months) | Hempel (76) |
| King et al. (56) | 2020 | Australia | Retrospective review | 98 infants | Gender: number (%)   - 39 (57.4) males < 29 wks or < 1000g - 16 (57.1) males >29 wks with evidence of brain injury - 29 (42.6) females <29 wks or < 1000g - 12 (42.9) >29 wks with evidence of brain injury   GA: mean (SD)   - 68 (70.8%) 27 wks (3.3) - 28 (29.2%) 37 wks (5.8)   BW (g): mean (SD)   - 879.5 (329.5) <29 wks or <1000g - 2537 (1461) > 29 weeks with evidence of brain injury   NE: n.s.  CP: not clearly stated  Therapeutic hypothermia: 13 (46.4%) term HIE | 2019 - 2020 | Medical history, ± cranial US, ± MRI | Prechtl | 12- 14 weeks postnatal age | At various ages of referral, by 1 year | n.s. |
| Morgan et al. (57) | 2016 | Australia | Prospective longitudinal and  cross-sectional study | 187 | Gender: distribution n.s. to maintain anonymity in a small hospital site  GA:   - term: 134 - late-preterm: n.s.   BW: n.s.  Late-preterm: n.s.  NE: 19  CP: 40  Therapeutic hypothermia: 0 | 2011 - 2013 | Medical history  and/or neuroimaging | Prechtl | 10 - 20 wks post term age | 12–24 months post term age - mean age 8.5 months (SD  = 4 months) | Neurological examination, clinical history and developmental  motor assessment |
| Table 4 continued | | | | | | | | | | | |
| Article | **Date** | **Country** | **Type of study** | **Population size** | **Population**  **(general characteristics)** | **Period of study** | **High risk identification** | **GMA** | **Age at GMA** | **Age of CP diagnosis** | **Method used for**  **neurological examinations** |
| Morgan et al. (58) | 2019 | Italy | Retrospective case controlled | 441 | Gender: Female (%)   - Normal: 52 - Mild disability: 1 - CP: 48   GA: < 32 weeks  Normal: 44  Mild disability: 42  CP: 44  32-36 weeks  Normal: 63  Mild disability: 65  CP: 63  >37 weeks:  Normal: 40  Mild disability: 40  CP: 40  Late preterm: n.s.  BW: n.s.  NE: HIE and perinatal asphyxia included but n.s. by GA  CP: yes, but n.s. by GA  Therapeutic hypothermia: n.s. | 2003-2014 | Exam and neuroimaging | Combined 3 month GMA by Prechtl, HINE (27) | Described as writhing and fidgety period up to 12 weeks post term | By 2 years of age | HINE (27) , Clinical Developmental Assessment (77) |
| Øberg et al. (59) | 2015 | Norway | Prospective cohort | 87 participants and 86 non-participants | Gender: 41 males (47%), 46 females (53%)  GA:   - preterm: 28.1-36.9 wks 46% (40) - ≥ 37 wks: 16% (14) - late preterm n.s. separately   BW: 1,528 g, (SD 1,045)  NE: 25  CP: 10 (12%)  Therapeutic hypothermia: 0 | Nov 3,  2002 - Oct 11, 2010 | Medical history | Prechtl | 3  months of age | 24 months | History and examination, definition by Rosenbaum et al. (1) |
| Table 4 continued | | | | | | | | | | | |
| Article | **Date of publication** | **Country** | **Type of study** | **Population size** | **Population**  **(general characteristics)** | **Period of study** | **High risk identification** | **GMA** | **Age at GMA** | **Age of CP diagnosis** | **Method used for**  **neurological examinations** |
| Prechtl et al. (13) | 1997 | Austria | Prospective cohort | 130 high and low-risk | Gender: 72 males (60%), 52 females (52%)  GA: 26-41wks (median 32 wks  -high-risk: 60 (46%)  -low -risk: 70 (64%))  -late-preterm: n.s. separately  BW (g) median (range):  - 700-4680g (median 1660g)  NE: n.s. separately  CP:   - high-risk: 45 - low -risk: 6   Therapeutic hypothermia: 0 | n.s. | Repeated cranial US | Prechtl | Preterm: 1^st^ - weekly until EDD  Term: 1^st^ -birth  2^nd^ (both term and preterm) – every 3-4 wks until 6-20 wks | By 24 months of age | Amiel-Tison and Grenier**(69)**  Bayley scale (71) and Griffiths Scales (72) |
| Seme-Ciglenecki (60) | 2003 | Slovenia | Prospective cohort | 132 high-risk preterms divided into 2 arms for assessment (high-risk and control) | Gender: 111 males (47.8%), 121 females (52.1%)  Boys/girls:   - high-risk: 56/64 - control: 55/57   112 age-matched low-risk controls  GA: <37wks median(range)  -high-risk: 33 (26-37)  -control:34 (24-37)  -late-preterm: n.s. separately  BW (g) median (range):  -high-risk: 1.975 (660-3.820)  -control: 1.930 (600-3.680)  NE: n.s. separately  CP:   - high-risk: 31 - control: 33   Therapeutic hypothermia: 0 | Oct 1, 1994 - Dec 31, 2000 | Random number table for selection amongst high-risk admissions | Prechtl | 3 months corrected age | 24 months corrected age | Amiel-Tison and Grenier**(69)** and Illingworth examinations (78) |
| Soleimani et al. (61) | 2015 | Iran | Case series | 15 late-preterm ≥ 35 wks and term | Gender: 8 males (53.3%), 7 females (46.7%)  GA: mean 37.3 ± SD 1.1  BW: mean 2800 ± SD 234  NE: 15 (12 Sarnat II, 3 Sarnat III)^22^  Abnormal neurological outcome (not definitely designated as CP): 10  Therapeutic hypothermia: 0 | 2012-2013 | Medical history | Prechtl | 3-5 months | 12-18 months age | Infant Neurological International Battery test (INFANIB) (81) |
| Stoen et al. (62) | Oct 2019 | Norway | Observational study | 405 | Gender: 54.3% male, 45.7% female  GA: mean 26.2 wks; SD 1.7 wks  Late preterm: n.s.  BW: mean 826g; SD 183g  NE: 57 (13.8%), n.s. by GA  CP: 42 (10.4%), n.s. by GA  Therapeutic hypothermia: n.s | 2009-2014 | Undefined | Prechtl | 10 – 15 wks post-term age | By mean age 3 years and 1 month | Surveillance of cerebral palsy in Europe (SCPE) (73) |
| Table 4 continued | | | | | | | | | | | |
| Article | **Date of publication** | **Country** | **Type of study** | **Population size** | **Population**  **(general characteristics)** | **Period of study** | **High risk identification** | **GMA** | **Age at GMA** | **Age of CP diagnosis** | **Method used for neurological examinations** |
| Sustersic et al. (63) | 2008 | Slovenia | Prospective cohort | 45 | Gender: 23 males (51.1%), 22 females (48.9%)  GA:  - 23-36 wks (mean,  31.6 weeks; SD, 3.3 wks)  - late-preterm: n.s.  BW: 525-3240 g (mean,  1788 g; SD, 718 g)  NE: 12 (27%)  CP: 6  Therapeutic hypothermia: 0 | Jan 2002 – Mar 2004 | Consecutive referrals and medical history | Prechtl | Term - 20 wks of post-term  age | 24 months | Amiel-Tison and Gosselin (69) |
| van Iersel et al. (64) | 2010 | Netherlands | Prospective cohort | 51  (17 preterm high-risk and 34 low-risk) | Gender: matched for gender but gender distribution n.s.  34 matched preterm controls  GA:   - <35 wks PMA - late-preterm 34 to 35 wks: n.s.   Median (range, wks):   - study group 32 (28–34) - control group 32 (28–34)   BW, g (± SD):   - study group 1817 (±611) - control group 1886 (±385)   NE: 17  CP:   - study group 2 (11%) - control group 2 (11%)   Therapeutic hypothermia: 0 | Jun 1999 - Jun 2007 | Medical history | Hadders-Algra | “preterm” (around 34 weeks  PMA), “writhing” (around term age) and “fidgety” GM age (around 3 months post term). | 18 months corrected age | TINE 's criteria (70) |
| Table 4 continued | | | | | | | | | | | |
| Article | **Date of publication** | **Country** | **Type of study** | **Population size** | **Population**  **(general characteristics)** | **Period of study** | **High risk identification** | **GMA** | **Age at GMA** | **Age of CP diagnosis** | **Method used for examinations** |
| Yang et al. (65) | 2012 | China | Longitudinal study | 79 | Gender: 60 males (75.9%), 19 females (24.1%)  Total: term: 47 (59.5%)  Male: female ratios:   - preterm 23:9 (72%:28%) - term 37:10 (79%:21%) n.s.   GA, wks (median):   - preterm: 32 (P25–P75=30–34) - term: 39 (P25–P75=38–40)   BW, g (mean):   - preterm: 1548 (SD=549) - term: 3200 (SD=423)   NE:   - preterm: 1 (3%) - term: 11 (23%)   CP: Total 65   - preterm: 29 (45%) - term: 36 (55%)   Therapeutic hypothermia: 0 | Sep 2003 - May 2009 | History and examination | Prechtl | 9 -20 wks post-term age | 2-5 years | History and examination, definition of Rosenbaum et al. (1) |
| Zhang et al. (66) | 2021 | China | Case control | 71 | Gender: 38 males (53.5%), 33 (46.5%) females  GA, wks:   - <37: 23 - ≥37: 48   BW, g:  NE: 8 with HIE  CP: 3 (4.23%)  Therapeutic hypothermia: | 2016 - 2018 | History and examination | Prechtl | 4-12 wks corrected GA | 1 year | Neurological examination and Gesell score (75) |
| Pilot studies/Protocols | | | | | | | | | | | |
| Pouppirt et al. (67) | 2021 | USA | Pilot prospective cohort study | 29 | Gender: 16 male (61.5%), 13 female (38.5%)  GA: ≥ 34 wks, 39 weeks (IQR 36.6, 40.1)  BW: median 3000 g (IQR 2610, 3520)  NE: All with HIE   - 7 mild - 13 moderate - 9 severe   CP:  Therapeutic hypothermia: 18   - 2 (29%) mild HIE - 9 (69%) moderate HIE   7 (78%) severe HIE | 2016 - 2018 | History and Sarnat assessment | Possibly Prechtl | First: Prior to anticipated hospital discharge or  by 30 days of life, (whichever came first)  Second: between 12  and <18 wks postterm age | Pending |  |
| Toldo et al. (68) | 2020 | India | Observational cohort study | Planned 1261 for predictive power of 0.95 and effect-size of 0.15 using Cohen’s f^2^ | GA: all GA | 2019 - 2021 | History and examination | Prechtl | 1-5 months | 12-24 months  and again at 3- 5 years |  |
| *Note*. BSID III = Bayley scales of infant and toddler development, 3rd edition, BW = birth weight, CP = cerebral palsy, EDD = Expected Date of Delivery, EEG = electroencephalogram, ELBW = extremely low birth weight), g = grams, FM = fidgety movements, GA = gestational age, GMA = general movements assessment, GMFCS = Gross Motor Function Classification Scale, HIE = hypoxic ischemic encephalopathy, HINE = Hammersmith Infant Neurological Examination, IQR = interquartile range, LBW = low birth weight, MOS = motor optimality score, Movement ABC = Movement Assessment Battery for Children, NBW = normal birth weight, NE = neonatal encephalopathy, n.s = not stated, PMA = postmenstrual age, SD = standard deviation, TINE= Touwen Infant Neurological Examination VLBW (very low birth weight), wks = weeks | | | | | | | | | | | |

| Table 5  *Key findings and limitations of excluded studies for the GMA and the predictive ability for CP in late-preterm and term infants with NE* | | | | | | | | |
| --- | --- | --- | --- | --- | --- | --- | --- | --- |
| **Article** | **Key findings with respect to GMA and CP** | **Predictive value of GMA** | | | | | **Limitations identified by the authors** | **Summarized reasons for exclusion** |
|  |  | **Sensitivity** | **Specificity** | **PPV** | **NPV** | **Other correlations** |  |  |
| Adde et al.^50^ | GM can strongly predict the development of CP in a clinical setting in both high-risk infants and identified infants that did not develop CP | 100%  (95% CI 73-100%) | 98%  (95% CI 91-99%) | - | - | - | Health professionals that followed up the low-risk group may not have been trained to detect subtle neurological changes and so a lower detection rate for CP may have occurred | Late-preterm infants were included in this study but not delineated as a group for their specific outcomes |
| Brogna et al.^51^ | GM- done in the fidgety period have a high sensitivity and specificity for CP prediction.  Trajectories of GM are also important predictors of outcomes, in that, consistently normal  GM trajectories lead into normal outcomes (95%). Transient GM abnormalities  (Abnormal–Normal trajectory) can either progress to a normal outcome (72%) or to a motor deficit (28%) | For CP: In the writhing period | | | | | Not stated | NE was not clearly identified as a diagnosis in this study although it was stated that patients were from a high-risk population |
|  |  | 100% | 86% |  |  | Spearman rank correlation  rs 0.68  (p < 0.001) |  |  |
|  |  | For CP: In the fidgety period | | | | |  |  |
|  |  | 100% | 97% |  |  | Spearman rank correlation  rs 0.78  (p < 0.001) |  |  |
|  |  | For CP: For the trajectories of GM | | | | |  |  |
|  |  | 100% | 97% |  |  | Spearman rank correlation  rs 0.69  (p < 0.001) |  |  |
| Table 5 continued | | | | | | | | |
| **Article** | **Key findings with respect to GMA and CP** | **Predictive value of GMA** | | | | | **Limitations identified by the authors** | **Summarized reasons for exclusion** |
|  |  | **Sensitivity** | **Specificity** | **PPV** | **NPV** | **Other correlations** |  |  |
| Cioni et al.^52^ | For all ages assessed, GMA highly correlated with neurological outcome and sensitivity and specificity was slightly higher than neurological observation.  Consistent PR pattern has more than 50% correlation with CP.  False positives and negatives were higher with neurological examination | Consistent GM abnormalities (PR or CS) as a strong predictor of an unfavorable outcome:  Sensitivity 88.9% | Consistently normal or transiently abnormal GM generally predict normal  development:  Specificity 95% | - | - | Range of agreement: 78-83%, between neurological and GMA was between | None stated | Term infants not separated by diagnosis so unsure of the outcomes related to NE |
| Dekkers et al. ^53^ | Children without anomalies on the MRI before hospital discharge  and normal movement quality at 3 months of age showed normal neurodevelopment at the age of  5  Individual motor trajectories show variability over time.  Presence of abnormal  GMs tend to detect CP and developmental problems | - | - | - | - | 12 % developed CP | Single-center study, with a small sample size resulting in limited power  Blinding the developmental assessments was not possible  Loss to follow-up occurred because of participation elsewhere in rehabilitation programs  Early physiotherapy treatment before 5 years was institutes and the impact of this on the motor performance trajectories of the individual children is difficult to determine | CP diagnosis at 2 years not clearly delineated although assessed, instead given at 5 years. |
| Dimitrijević et al.^54^ | GM that are CS are highly predictive of CP, but not the same for the PR pattern | 100% | 72.1% | - | - | - | External validity is limited by a small population size with PR movements. Neuroimaging data not available for all patients | Late-preterm infants were included in this study but not delineated as a group for their specific outcomes. |
| Einspieler et al.^55^ | Sporadic FM indicate adverse neurodevelopmental outcome but not necessarily CP | 15% of infants who later developed CP had sporadic FM which was linked to a slightly better (although not normal) concurrent movement repertoire | - | - | - | - | There was a small sample of only 9, for those with sporadic FM.  Only high-risk groups were used and so no comparator for sporadic FM available from general population | Term and late-preterm infants were included in this study but not delineated as a group for their specific diagnosis of NE nor for their specific outcomes.  Computer based analysis was used for coding of temporal organization of FM |

| Table 5 continued | | | | | | | | |
| --- | --- | --- | --- | --- | --- | --- | --- | --- |
| **Article** | **Key findings with respect to GMA and CP** | **Sensitivity** | **Specificity** | **PPV** | **NPV** | **Other correlations** | **Limitations identified by authors** | **Summarized reasons for exclusion** |
| Einspieler et al.^56^ | In children with CP:   - 95% did not have FM - 100% had a non-optimal MOS - GMFCS level was strongly correlated to MOS - An MOS > 14 was most likely associated with GMFCS outcomes I or II, whereas GMFCS outcomes IV or V were hardly ever associated with an MOS > 8   A number of different movement patterns were associated with more severe functional impairment (GMFCS III–V | - | - | - | - | - | Variable:  - access to prenatal care  - neonatal intensive care managements  - environmental factors such as socio-economic status, teratogens,  etc.  - varied ethnic backgrounds  The number of individuals varied compared to the usual occurrence rate:  - dyskinesia overrepresented  -ataxia and hypotonia  underrepresented  Heterogeneous local settings for assessment of GM videos | Sensitivity, specificity, PPV and NPV not calculated specifically  Term GA not differentiated from preterm for outcome |

| Table 5 continued | | | | | | | | | | | | | | | | | | | | | |
| --- | --- | --- | --- | --- | --- | --- | --- | --- | --- | --- | --- | --- | --- | --- | --- | --- | --- | --- | --- | --- | --- |
|  | | | | | | | | | | | | | | | | | | | | | |
| **Article** | **Key findings with respect to GMA and CP** | | | **Predictive value of GMA** | | | | | | | | | | | | | | | **Limitations identified by the authors** | | **Summarized reasons for exclusion** |
|  |  |  |  | **Sensitivity** | | **Specificity** | | | **PPV** | | | | **NPV** | | | **Other correlations** | | |  |  |  |
| Feng et al.^57^ | Both the qualitative assessment of GM and EEG examination can be used to predict high-risk neonatal adverse neurodevelopmental outcome with the combination having higher sensitivity, specificity, PPV and NPV | | | Prediction of CP of PR movements | | | | | | | | | | | | | | | Not stated | | Term and late-preterm infants were included in this study but not delineated as a group for their specific diagnosis of NE nor for their specific outcomes |
|  |  |  |  | 25% | | | 68.9% | | | 2.9% | | 96% | | | - | | | |  |  |  |
|  |  |  |  | Prediction of CP of CS movements | | | | | | | | | | | | | | |  |  |  |
|  |  |  |  | 50% | | | 98.1% | | | 50% | | 98.1% | | | - | | | |  |  |  |
|  |  |  |  | Prediction of CP of absent FM | | | | | | | | | | | | | | |  |  |  |
|  |  |  |  | 75% | | | 99% | | | 75% | | 99% | | | - | | | |  |  |  |
|  |  |  |  | Prediction of CP of combined GM and EEG | | | | | | | | | | | | | | |  |  |  |
|  |  |  |  | 90.48% | | | 95.45% | | | 86.36% | | 96.92% | | | - | | | |  |  |  |
|  |  |  |  | For predicting high-risk neonatal neurodevelopmental outcome | | | | | | | | | | | | | | |  |  |  |
|  |  |  |  | 83.87% | | | 84.81% | | | 68.42% | | 93.06% | | | - | | | |  |  |  |
|  |  | | |  | | | | | | | | | | | | | | |  | |  |
| Goyen et al. ^58^ | Excellent predictive validity for the clinical use of the GMA at 3 months in  relation to 2–3-year outcomes  Excellent levels of sensitivity  and specificity for detecting CP were  maintained in the clinical setting | | | For absent/abnormal fidgety movements (95% CI)   \| 97.6%  (85.6–99.9) \| 95.7%  (90.4–98.2) \| \| --- \| --- \| | | | | | | | | | | | | | | | A convenience sample was used in the study  The age for developmental outcome varied between 2 and 3 years  26% were lost to follow or discharged from clinical service prior to 2 years | | CP outcomes not delineated into preterm versus term but given as a combined outcome |
| Guzetta et al.^59^ | Confirms the high sensitivity and specificity, both during writhing and fidgety periods, of the Prechtl’s method of assessment of GM based on video observation. The results also support the use of GM by direct assessment when the full application of the standard video observation is not routinely possible | | | Writhing period GMA | | | | | | | | | | | | | | | The level of expertise of the examiners may have been higher than average, therefore making the findings not  replicable in other contexts | | Term and late-preterm infants were included in this study but not delineated as a group for their specific diagnosis of NE. Late-preterms were also not separated from other preterms for their outcomes |
|  |  |  |  | 100% | | | 84% | | | 43% | | 100% | | | - | | | |  |  |  |
|  |  |  |  | Fidgety period GMA | | | | | | | | | | | | | | |  |  |  |
|  |  |  |  | 100% | | | 95% | | | 58% | | 100% | | | - | | | |  |  |  |
| Table 5 continued | | | | | | | | | | | | | | | | | | | | | |
| **Article** | **Key findings with respect to GMA and CP** | | | **Predictive value of GMA** | | | | | | | | | | | | | | | **Limitations identified by the authors** | | **Summarized reasons for exclusion** |
|  |  |  |  | **Sensitivity** | | **Specificity** | | | **PPV** | | | | **NPV** | | | **Other correlations** | | |  |  |  |
| Hadders-Algra et al.^60^ | GM quality after 47 wks PMA was  strongly related to neurodevelopmental outcome at l 1/2 years of age, suggesting that the absence of the age-specific ‘fidgety’ character of GM could be a herald of disability | | | Neurological outcome of CP and mental retardation | | | | | | | | | | | | | | | Not stated | | Outcomes not delineated into neuromotor outcomes of CP but given as a combined outcome with mental retardation |
|  |  |  |  | 88% | | | 88% | | | 88% | | 88% | | | - | | | |  |  |  |
|  |  |  |  |  | | |  | | |  | |  | | |  | | | |  |  |  |
| King et al. ^61^ | Absent fidgety movements are highly predictive of CP in both the preterm and term infant  94% with absent fidgety movements diagnosed with CP or high risk of CP | | | - | | | - | | | - | | - | | | - | | | | Small sample size limited statistical subgroup analysis  Telehealth appointments and crying infant states were barriers to performing the HINE and GMA in some infants. | | Sensitivity, specificity, PPV and NPV not calculated specifically |
| Morgan et al.^62^ | GMA had excellent sensitivity and specificity to predict infants who would later be diagnosed with CP as well as those with normal outcomes. One benefit of early detection  using GMA was that diagnosis occurred earlier, on average  at 8.5 months in the study compared to 17 months in their CP registry | | | 98%  (95% CI 86.79–99.58) | | | 94%  (95% CI 88.69–97.16) | | | - | | - | | | - | | | | Sampling bias was possible, as all infants in the study were already considered at high-risk of adverse neurodevelopmental outcome. Outcome data were mostly only at 12 months but milder forms of CP may be first diagnosed later in childhood when the motor impairment was deemed as definitely permanent. There was practice variation between sites in terms of number of blinded GM scorers although they stated that there was no scoring accuracy differences | | Term infants were included in this study but not delineated as a group for their specific diagnosis of NE as it related to their specific outcomes |
| Table 5 continued   \| **Article** \| **Key findings with respect to GMA and CP** \| **Predictive value of GMA** \| \| \| \| \| **Limitations identified by the authors** \| **Summarized reasons for exclusion** \| \| --- \| --- \| --- \| --- \| --- \| --- \| --- \| --- \| --- \| \| **Sensitivity** \| **Specificity** \| **PPV** \| **NPV** \| **Other correlations** \| | | | | | | | | | | | | | | | | | | | | | |
| Morgan et al.^63^ | In a routine clinical setting, the GMA strongly predicts neurodevelopmental impairments at 2 years in high-risk infants  FM assessment proves to be a valuable tool for detecting subsequent motor problems early in life when performed in a routine hospital clinical practice. The risk of developing motor problems by the age of 2 years increases linearly with the extent of abnormal FM findings. This risk is also 10 times higher if FM are absent by 3 months of age than if FM are normal. | | | When absent FM were considered to be a positive test result (and normal, abnormal, or sporadic FM were considered to be a negative test result) | | | | | | | | | | | | | | | Relatively small study sample.  Study sample  did not include all infants hospitalized  during the study period, only the high-risk which could introduce sampling bias | | Term and late-preterm infants were included in this study but not delineated as a group for their specific diagnosis of NE nor for their specific outcomes |
|  |  |  |  | 90%  (95% CI 56%, 100%) | | | 90%  (95% CI 81%, 95%) | | | 53% | | 99% | | | LR for a positive test result:  8.7 (95% CI 4.4, 17.2)  LR for a negative test result:  0.1 (95% CI 0, 0.7) | | | |  |  |  |
|  |  |  |  | When any finding but normal (normal FM) was considered to be a positive test result | | | | | | | | | | | | | | |  |  |  |
|  |  |  |  | 100%  (95% CI69%, 100%) | | | 70%  (95% CI59%, 80%) | | | 30% | | 100% | | | LR for positive test result:  3.3 (95% CI 2.4, 4.7)  LR for a negative test result: 0 (95% CI 0, 1.1) | | | |  |  |  |
|  |  |  |  |  | |  | | |  | | | |  | | |  | | |  |  |  |
| Table 5 continued | | | | | | | | | | | | | | | | | | | | | |
| \| **Article** \| **Key findings with respect to GMA and CP** \| **Predictive value of GMA** \| \| \| \| \| **Limitations identified by the authors** \| **Summarized reasons for exclusion** \| \| --- \| --- \| --- \| --- \| --- \| --- \| --- \| --- \| --- \| \| **Sensitivity** \| **Specificity** \| **PPV** \| **NPV** \| **Other correlations** \| | | | | | | | | | | | | | | | | | | | | | |
| Morgan et al. cont’d ^63^ | Pooled predictive power of early GMA, neuroimaging, plus HINE was higher than the 3 tools in isolation | | | Using GMA only | | | | | | | | | | | | | Retrospective data: impacts accuracy and generalizability of results    Selection bias: Recruitment is exclusively from hospitals  The individuals selected as controls may under-represent the population    Generalizability affected: Highly skilled assessors used  Imperfect interrater reliability of tests: lack of 100% agreement on the scoring of each of the 3 tests | | | | GA and NE not differentiated into categories the results |
|  |  |  |  | 95% | | 97% | | | - | | | | - | | | - |  |  |  |  |  |
|  |  |  |  | Combined predictive  power (GMA, HINE and neuroimaging) | | | | | | | | | | | | |  |  |  |  |  |
|  |  |  |  | 97.86% | | 99.22% | | | 98.56% | | | | 98.84% | | | - |  |  |  |  |  |
|  |  |  |  |  | |  | | |  | | | |  | | |  |  |  |  |  |  |
| Table 5 continued | | | | | | | | | | | | | | | | | | | | | |
| **Article** | **Key findings with respect to GMA and CP** | | | **Predictive value of GMA** | | | | | | | | | | | | | | | **Limitations identified by the authors** | | **Summarized reasons for exclusion** |
|  |  |  |  | **Sensitivity** | | **Specificity** | | | **PPV** | | | | **NPV** | | | **Other correlations** | | |  |  |  |
| Oberg et al.^64^ | In a routine clinical setting, the GMA strongly predicted neurodevelopmental impairments at 2 years in high-risk infants | | | For absent FM | | | | | | | | | | | | | | | Only high-risk infants were assessed which could be a source of bias  Limited number of assessors used but disadvantage was the effect of fatigue on the assessments | | Late-preterm infants were included in this study but not delineated as a group for their specific outcomes |
|  |  |  |  | 90%  (95% CI, 56 - 100) | | 90%  (95% CI=81%, 95%) | | | 53% | | | | 99% | | | LR for a positive test result was 8.7 (95% CI=4.4, 17.2), LR negative test result was 0.1 (95% CI=0, 0.7) | | |  |  |  |
|  |  | | | For any finding but normal (normal FM) | | | | | | | | | | | | | | |  | |  |
|  |  | | | 100% (95% 0=69%, 100%), | | 70% (95% 0 = 59%, 80%) | | | 30% | | | | 100% | | | LR for positive and negative test results were 3-3 (95% 0 = 2.4, 4.7) and 0 (95% 0=0, 1.1) | | |  | |  |
| Prechtl et al.^23^ | The Prechtl technique for assessing spontaneous motor activity can identify and distinguish between those infants who require early intervention for neurological abnormalities and those who do not | | | 95% | | 96% | | | - | | | | - | | |  | | | None identified | | GA not delineated for the outcomes  NE not delineated in the risk factors  CP not distinguished in the neurological outcomes |
| Seme-Ciglenecki et al.^65^ | GMA have better validity, sensitivity, specificity, PPV,  and NPV when compared to the classical neurological examination of preterm infants that are high-risk | | | For GMA | | | | | | | | | | | | | | | In Slovenia GM are not routinely used limiting the comparison of  results of the study and the results of other investigators, that is limiting control of interscorer agreement | | Late-preterm infants were included in this study but not delineated as a group for their specific outcomes |
|  |  |  |  | 94% | | | 92% | | | 81% | | 98% | | | Validity  92% | | | |  |  |  |
|  |  |  |  | For classical neurological assessment | | | | | | | | | | | | | | |  |  |  |
|  |  |  |  | 60% | | | 97% | | | 43% | | 44% | | | 97% | | | |  |  |  |
| Soleimani et al.^66^ | In children born at term GA  with neonatal HIE, FM- assessment improves the ability to predict later neurodevelopmental outcomes | | | The predictive values of FM: Neurodevelopmental outcomes of abnormal FM (mild to moderate and moderate to severe HIE) | | | | | | | | | | | | | | | Recurrent assessments and long-term follow-ups may have been a limitation for families. Neuroimaging was limited to limited resources | | The outcomes were described in the terms “neurodevelopmental outcomes” as the authors considered it not possible to diagnose CP definitively at 12 - 18 months |
|  |  |  |  | 80  (95% CI, 44 - 96) | | 100  (95% CI, 47 -100) | | | - | | | | - | | | Accuracy  87 (95% CI, 57 to 100)  Cramer's V 0.661 | | |  |  |  |
|  |  |  |  | The predictive values of FM: Neurodevelopmental outcomes of abnormal FM (meaning moderate to severe HIE only) | | | | | | | | | | | | | | |  |  |  |
|  |  |  |  | 83.3  (95% CI, 35.9 - 99.6) | | 66.7  (95% CI, 29.9 - 92.5) | | | - | | | | - | | | Accuracy 73.3 (95% CI, 42.8 to 94.4)  Cramer's V 0.491 | | |  |  |  |
| Stoen et al.^67^ | Predictive accuracy of GMA increased when sporadic FM not categorized as a marker for later CP  Absence of FM correctly predicted CP in about 50% of cases  Neonatal cerebral imaging in combination with GMA increased the predictive accuracy | | | Absent/sporadic FM | | | | | | | | | | | | | | | Longer follow-up might have resulted in more children presenting  with a mild CP phenotype  being diagnosed with CP and, consequently, poorer performance  of GMA | | Late-preterm infants were included in this study but not delineated as a group for their specific outcomes  CP diagnosis done past 2 years of age |
|  |  |  |  | 69.1  (95% CI 52.9–82.4) | | 91.5  (95% CI 88.1–94.1) | | | 48.3  (95% CI 38.7–58.1) | | | | 96.2  (95% CI 94.2–97.6) | | | Accuracy % (CI 95%)  89.1  (85.7–92.0) | | |  |  |  |
|  |  |  |  | Abnormal neonatal imaging | | | | | | | | | | | | | | |  |  |  |
|  |  |  |  | 81.0  (95% CI 65.9–91.4) | | 85.3  95% CI 81.2–88.8) | | | 39.1  (95% CI 32.5–46.1) | | | | 97.5  (95% CI 95.4–98.6) | | | Accuracy %  (CI 95%)  84.9  (81.0–88.2) | | |  |  |  |
|  |  |  |  | Absent/sporadic FM and/or abnormal imaging | | | | | | | | | | | | | | |  |  |  |
|  |  |  |  | 88.1  (95% CI 74.4–96.0) | | 70.3  (95% CI 65.3–75.0) | | | 25.7  (95% CI 22.3–29.6) | | | | 98.1  (95% CI 95.7–99.1) | | | Accuracy %  (CI 95%)  72.1 (67.5–76.5) | | |  |  |  |
|  |  |  |  | Absent/sporadic FM and abnormal imaging | | | | | | | | | | | | | | |  |  |  |
|  |  |  |  | 61.9  (95% CI 45.6–76.4) | | 94.2  (95% CI 91.3–96.4) | | | 55.3  (95% CI 43.4–66.6) | | | | 95.5  (95% CI 93.5–96.9) | | | Accuracy %  (CI 95%)  90.8 (87.6–93.5) | | |  |  |  |
|  |  |  |  | Absent FM and/or abnormal imaging | | | | | | | | | | | | | | |  |  |  |
|  |  |  |  | 88.1  (95% CI 74.4–96.0) | | 77.6  (95% CI 72.9–81.8) | | | 31.4  (95% CI 26.8–36.3) | | | | 98.3  (95% CI 96.1–99.2) | | | Accuracy %  (CI 95%)  78.7 (74.3–82.6) | | |  |  |  |
|  |  |  |  | Absent FM and abnormal imaging | | | | | | | | | | | | | | |  |  |  |
|  |  |  |  | 61.9  (95% CI 45.6–76.4) | | 99.2  (95% CI 97.6–99.8) | | | 89.7  (95% CI 73.3–96.5) | | | | 95.7  (95% CI 93.8–97.1) | | | Accuracy %  (CI 95%)  95.3 (92.7–97.1) | | |  |  |  |
| Sustersic et al.^68^ | Confirmed the importance of abnormal  GM at ages 2-4 months in predicting  CP | | | Correlation between GMA and neurologic outcome at term age | | | | | | | | | | | | | | | The study group was relatively small and heterogenous in terms of risk factors for brain damage. Compared with previous studies, the group were much younger at  time of evaluation of neurologic state | | Late-preterm infants were included in this study but not delineated as a group for their specific outcomes |
|  |  |  |  | - | | | - | | | - | | - | | | Pearson’s R  0.51 | | | |  |  |  |
|  |  |  |  | Correlation between neurologic outcome and assessment during the FM period | | | | | | | | | | | | | | |  |  |  |
|  |  |  |  |  | | |  | | |  | |  | | | Pearson’s R  0.50  (in the group of children with minimal CP) | | | |  |  |  |
| Table 5 continued | | | | | | | | | | | | | | | | | | | | | |
| **Article** | **Key findings with respect to GMA and CP** | | | **Predictive value of GMA** | | | | | | | | | | | | | | | **Limitations identified by the authors** | | **Summarized reasons for exclusion** |
|  |  |  |  | **Sensitivity** | | | **Specificity** | | | **PPV** | | **NPV** | | | **Other correlations** | | | |  |  |  |
| van Iersel et al.^69^ | Perinatal asphyxia in preterm infants is not associated with an increased risk for neurodevelopmental  problems including CP  Respiratory problems during the neonatal period are associated with PVL and  adverse neurological outcome | | | - | | | - | | | - | | - | | | Chi square for  Trend, p=0.001  Quality of GM at “preterm” and “writhing” age were not related to the development of CP  The quality of GMs at FM age was related to the development of CP | | | | Limited access to neuro-imaging.  Inherent to the limitations of the setting, only information on the ultrasound scans were available. A small sample size was obtained as the institution was not a referral center for neonates with asphyxia | | Late-preterm infants were included in this study but not delineated as a group for their specific outcomes |
| Yang et al.^70^ | Later functional limitations in children with CP can be predicted with the aid of the assessment of the quality of motor performance at 9 - 20 wks post-term age (irrespective of the GA) | | | The association between the quality of movements at 9 - 20 weeks post-term age and the GMFCS levels | | | | | | | | | | | | | | | Participants are a sample of convenience:  inclusion criteria were that their motor performance was videoed  around their 4th month post-term age and that they had developed  CP | | Late-preterm infants were included in this study but not delineated as a group for their specific outcomes |
|  |  |  |  | - | | | - | | | - | | - | | | Spearman correlation coefficient −0.52 (p<0.001). | | | |  |  |  |
|  |  |  |  | The association between the postural patterns found at 9 to 20 weeks post-term age and the children's later GMFCS levels | | | | | | | | | | | | | | |  |  |  |
|  |  |  |  | - | | | - | | | - | | - | | | Kendall-Tau-c −0.19  (p<0.05) | | | |  |  |  |
|  |  |  |  | The association between a CS movement character and the later GMFCS levels | | | | | | | | | | | | | | |  |  |  |
|  |  |  |  | - | | | - | | | - | | - | | | Kendall-Tau-c 0.41  (p<0.001) | | | |  |  |  |
| Zhang et al. ^71^ | | The combination of EEG, ERP, and GMs quality assessment can greatly improve the prediction of neurodevelopmental outcome of high-risk newborns.  Kappa statistic for the reliability of predicting  neurodevelopmental outcome of high risk newborns by the EEG+ERP+GMs method was substantial | GMA | | | | | | | | | | | | | | | Small sample size limits the power of the study  Multicenter studies, more accurate monitoring of brain  function, and longer developmental trials would provide  more support for the results of this study | | Population for term vs preterm not delineated in outcome. Outcome not delineated specifically as CP. | |
|  |  |  | 82.35% | | 83.33% | | | 83.33% | | | 83.33% | | | Kappa statistic  0.586 | | | |  |  |  |  |
|  |  |  | Combination of GMA+EEG+ERP | | | | | | | | | | | | | | |  |  |  |  |
|  |  |  | 90.00%, | | 95.08% | | | 75.00% | | | 98.31% | | | Kappa statistic 0.785 | | | |  |  |  |  |
| \| Pilot studies/Protocols – results pending \| \| \| \| \| \| \| \| \| \| --- \| --- \| --- \| --- \| --- \| --- \| --- \| --- \| --- \| \| Poupprit et al. ^72^ \| All degrees of hypoxic-ischemic encephalopathy had abnormal general movements during the writhing age, which normalized by the fidgety age. \|  \|  \|  \|  \|  \| Enrollment of infants with mild hypoxic-ischemic encephalopathy proved challenging as these infants are not always identified clinically, and the definition of mild hypoxic-ischemic encephalopathy varies widely.  Little is known about the effect of therapeutic hypothermia on an infant’s early spontaneous movements and these transient findings.  Small population size limits power  Differences in interrater reliability may also be due to examiner experience, despite undergoing formal General  Movements Assessment training \| Lack of long-term neurodevelopmental outcomes. \| | | | | | | | | | | | | | | | | | | | | | |
| \| Toldo et al. ^73^ \| Research objectives  (1) to relate the GMA to the outcome at 12–24 months. Research objective  (2) to investigate the impact of predefined exposures  (3) to evaluate the  interscorer agreement of GMA. \|  \|  \|  \|  \|  \| Measurement biases may occur because blinding is not possible  Higher-order impacts may be underestimated as a follow-up period of 24 months may be too short because certain neurological and developmental disorders cannot be diagnosed with absolute certainty at this age  Developmental delay, in particular, may occur after weaning from breast feeding because of undernourishment,  and might therefore not be detected by means of GMA during the first months of life \|  \| \| --- \| --- \| --- \| --- \| --- \| --- \| --- \| --- \| --- \| | | | | | | | | | | | | | | | | | | | | | |
| *Note*. CI = confidence interval, CP = cerebral palsy, CS = cramped-synchronized, EEG = electroencephalogram, ERP = Event related potential, FM = fidgety movements, GA = gestational age, GM = general movements, GMA = general movements assessment, GMFCS = gross motor function classification system, LR = likelihood ratio, MOS = motor optimality score, NE = neonatal encephalopathy, NPV = negative predictive value, PMA = postmenstrual age, PR = poor repertoire, PPV = positive predictive value | | | | | | | | | | | | | | | | | | | | | |

| Table 6  *General characteristics of identified studies for the GMA and the predictive ability for CP in late-preterm and term infants with NE* | | | | | | | | | | | |
| --- | --- | --- | --- | --- | --- | --- | --- | --- | --- | --- | --- |
| Article | **Date of publication** | **Country** | **Type of study** | **Population size** | **Population (general characteristics)** | **Period of study** | **Method of high-risk identification** | **GMA** | **Age at GMA** | **Age of CP diagnosis** | **Method used for neurological examinations** |
| Ferrari et al. (82) | 2011 | Italy | Case series | 34 term | Gender: 20 males (58.8%), 14 females (41.2%)  GA: mean 40.4 ± 1.2 wks  BW: mean 3536 9 (SD ± 457g)  NE: 34  CP: 16  Therapeutic hypothermia: 0 | 2003 -  2006 | History of HIE | Prechtl | At 1-3 postnatal months | 24 months | Amiel-Tison and Grenier (69) and an extension of TINE 's criteria (70) |
| Glass et al. (83) | 2021 | United States | Observational prospective cohort | 58 term | Gender: 27 males (47%), 31 females (53%)  GA: 39.55 wks (± 1.58)  BW: 3428g (± 594)  NE: 58, HIE 42 (71%)  CP: 7 (12%)  Therapeutic hypothermia: 40 (68%) | 2015 - 2017 | Clinical diagnosis ± EEG, ± MRI | Prechtl | Median of 15.4 weeks | 2- 3 years, 31 months (mean) | History on medical record, neurological examination and classified by GMFCS (74) |
| Prechtl et al. (84) | 1993 | Italy | Case series | 26 term | Gender: 8 males (30.7%), 18 females (69.2%)  GA: 37 – 41 wks (mean and SD not done)  BW: 2250 – 4150g (mean and SD not done)  NE: mild to moderate: 13  severe: 13  CP: 10  Therapeutic hypothermia: 0 | 1985 -1989 | Medical history | Prechtl | 0-22 wks | 17-24 months of age | Griffiths Scales (72) |
| *Note.* BW = birth weight, CP = cerebral palsy, GA = gestational age, GMA general movements assessment, EEG = electroencephalogram, GMFCS = gross motor function classification system, HIE = hypoxic ischemic encephalopathy, MRI = magnetic resonance imaging, NE = neonatal encephalopathy, n.s. = not stated, SD = standard deviation, TINE= Touwen Infant Neurological Examination, US = ultrasound, wks = weeks | | | | | | | | | | | |

| Table 7  *Key findings and limitations of included studies for the GMA and the predictive ability for CP in late-preterm and term infants with NE* | | | | | | | |
| --- | --- | --- | --- | --- | --- | --- | --- |
| **Article** | **Key findings with respect to GMA and CP** | **Predictive value of GM** | | | | | **Limitations identified by the authors** |
|  |  | **Sensitivity** | **Specificity** | **PPV** | **NPV** | **Other correlations** |  |
| Ferrari et al.^88^ | With HIE in term neonates, the site and severity of brain lesions seen on early MRI are highly correlated  with GM  Central gray matter damage leads to CS GM and poor motor outcome    For the prediction of motor outcomes, both early MRI scans and GM are complementary | For CS movements | | | | | Fewer grade 1 HIE infants may be present in the sample as not all HIE infants are referred to that tertiary center (not representative of the population)    MRI performed over first 6 postnatal wks (relatively wide period)  Very early MRI scans are not as reliable in predicting motor outcome versus when performed later  Transient abnormalities, such as cerebral edema, can lead to either overestimation or underestimation of the severity of damage |
|  |  | 100% | 68.7% | 100% | 78.3% | - |  |
| Glass et al. ^89^ | Normal neonatal MRI and GMA done at three months of age indicates that a high-risk term-born child is likely at low risk for moderate/severe CP | For absent FMs  (95% CI) | | | | | Low sample size limits the study’s power  In-person evaluations could not be standardized  Assessments done at 2-3 years may capture mild impairments that may resolve or may not capture all impairments that become obvious as the child ages, obviating the need for longer follow-up  The impact of early intervention could not be measured |
|  |  | \| 29%  (4-71%) for any CP  50%  (1-99%) for moderate to severe CP \| 98%  (90-100%) for any CP  96%  (89-100%) for moderate to severe CP \| 67%  (9 – 99%) for any CP  33%  (1-91%) for moderate to severe CP \| 90%  (79 – 96%) for any CP  98%  (90 – 100%) for moderate to severe CP \| Relative risk  7.3  (2.3-23.3)  Relative risk  18.3 (1.5-227.1) \| \| --- \| --- \| --- \| --- \| --- \| \| \| For absent myelination of the PLIC and absent FMs \| \| \| \| \| \| --- \| --- \| --- \| --- \| --- \| \| 14%  (0-58%) for any CP  50%  (1-99%) for moderate to severe CP \| 98%  (90%-100%) for any CP  96%  (89%-100%) for moderate to severe CP \| 67%  (9 – 99%) for any CP  33%  (1%-91%) for moderate to severe CP \| 90%  (79 – 96%) for any CP  98%  (90 – 100%) for moderate to severe CP \| Relative risk  7.3  (2.3-23.3)  Relative risk  18.3 (1.5-227.1) \| \| \| \| \| \| | | | | |  |
| Prechtl et al.^90^ | Changes in spontaneous motility and especially GM developmental trajectories  are good predictors of the neurological outcome  The predictive value of GMA is similar to that of EEG and neuro-imaging, and better than neurological  examination | Early observation (first 2 wks) | | | | | None stated |
|  |  | 100% | 46.2% | 65% | 100%. | GM quality and outcome correlation:  r = 0.61, (P < 0.001)  Neurological findings and outcome correlation:  r = 0.34, (P > 0.1) |  |
|  |  | Late observation (15-22 wks) | | | | |  |
|  |  | 84.6% | 84.6% | 84.6% | 84.6% | Both observation and neurological assessment are highly correlated with the  outcome and for both:  r = 0.88  (P < 0.001) |  |
| *Note.* CI = confidence interval, CP = cerebral palsy, CS = cramped synchronized, EEG = electroencephalogram, Fidgety Movements = FMs, GM = general movements, GMA = general movements assessment, HIE = hypoxic ischemic encephalopathy, MRI = magnetic resonance imaging, NE = neonatal encephalopathy, NPV = negative predictive value, PPV = positive predictive value, r = correlation coefficient, wks = weeks. | | | | | | | |
