# Supplementary material for: The assessment of general movements in term and late-preterm infants diagnosed with neonatal encephalopathy, as a predictive tool of cerebral palsy by 2 years of age—a scoping review
Source: Syst Rev. 2021 Aug 12;10:226. doi: 10.1186/s13643-021-01765-8 (PMC8359053; doi:10.1186/s13643-021-01765-8)

Additional File 5

Risk of Bias Assessment

| Table 8  JBI Critical Appraisal Checklist for Case Series | | |  |
| --- | --- | --- | --- |
| **JBI checklist questions** | **Ferrari et al. 2011**  **(82)** | **Prechtl et al. 1993 (84)** | **%** |
| Were there clear criteria for inclusion in the case series? | Yes | Uncertain | 50.0 |
| Was the condition measured in a standard, reliable way for all participants included in the case series? | Uncertain | Uncertain | 0.0 |
| Were valid methods used for identification of the condition for all participants included in the case series? | Yes | Yes | 100.0 |
| Did the case series have consecutive inclusion of participants? | Uncertain | Uncertain | 0.0 |
| Did the case series have complete inclusion of participants? | Uncertain | Uncertain | 0.0 |
| Was there clear reporting of the demographics of the participants in the study? | Yes | Yes | 100.0 |
| Was there clear reporting of clinical information of the participants? | Yes | Yes | 100.0 |
| Were the outcomes or follow up results of cases clearly reported? | Yes | Yes | 100.0 |
| Was there clear reporting of the presenting site(s)/clinic(s) demographic information? | Yes | Yes | 100.0 |
| Was statistical analysis appropriate? | Yes | Yes | 100.0 |


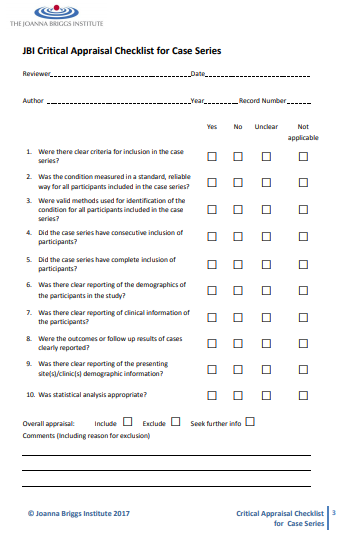


| Table 9  JBI Critical Appraisal Checklist for Cohort Studies | | |  |
| --- | --- | --- | --- |
| **JBI checklist questions** | **Glass et al. 2011**  **(83)** |  | **%** |
| Were the two groups similar and recruited from the same population? | Yes |  | 100.0 |
| Were the exposures measured similarly to assign people to both exposed and  unexposed groups? | Yes |  | 100.0 |
| Was the exposure measured in a valid and reliable way? | Yes |  | 100.0 |
| Were confounding factors identified? | Yes |  | 100.0 |
| Were strategies to deal with confounding factors stated? | Yes |  | 100.0 |
| Were the groups/participants free of the outcome at the start of the study (or at the  moment of exposure)? | Yes |  | 100.0 |
| Were the outcomes measured in a valid and reliable way? | Yes |  | 100.0 |
| Was the follow up time reported and sufficient to be long enough for outcomes to occur? | Yes |  | 100.0 |
| Was follow up complete, and if not, were the reasons to loss to follow up described  and explored?  Were strategies to address incomplete follow up utilized? | Yes  Unclear |  | 100.0  50.0 |
| Was statistical analysis appropriate? | Yes |  | 100.0 |


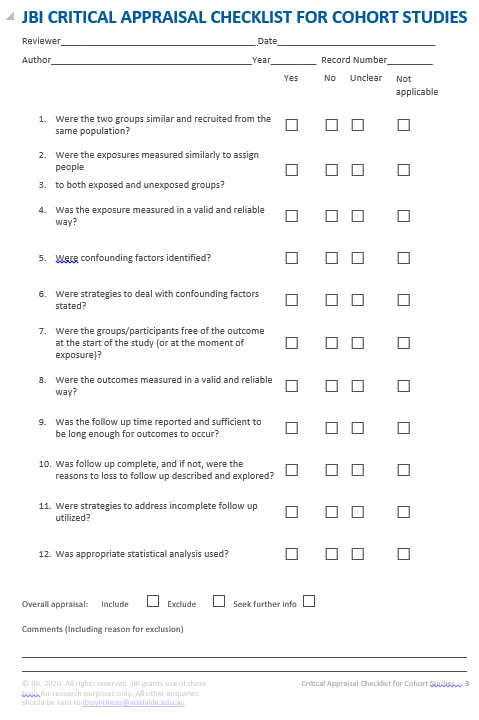

Supplement: Supplementary file 5 — Additional file 5. Critical appraisal. [file 13643_2021_1765_MOESM5_ESM.docx]
