# Supplementary material for: The assessment of general movements in term and late-preterm infants diagnosed with neonatal encephalopathy, as a predictive tool of cerebral palsy by 2 years of age—a scoping review
Source: Syst Rev. 2021 Aug 12;10:226. doi: 10.1186/s13643-021-01765-8 (PMC8359053; doi:10.1186/s13643-021-01765-8)
Supplement: Supplementary file 6 — Additional file 6. Tables for data extraction. [file 13643_2021_1765_MOESM6_ESM.docx]

Additional file 6

Tables for Data Extraction

Scoping review General movements assessment in late-preterm and term infants diagnosed with Neonatal encephalopathy, as a predictive tool of cerebral palsy by two years of age

Table 4 Data extraction

| Table 4  Key Findings and characteristics of studies for the GMA and predictive ability for CP in late-preterm and term infants with NE | | | | | | | | | | | | | | | | |  |
| --- | --- | --- | --- | --- | --- | --- | --- | --- | --- | --- | --- | --- | --- | --- | --- | --- | --- |
| Article | Date of publication | Country | Type of study | Population size (number of infants) | Population (general characteristics) | Period of study | | High risk identification | | | GMA | | Age at GMA | Age of CP diagnosis | | Method used for neurological examination |  |
|  |  |  |  |  |  |  |  | |  |  | |  | | |  | | |
|  |  |  |  |  |  |  |  | |  |  | |  | | |  | | |
| Note. CP = Cerebral palsy, GMA = General movements assessment | | | | | | | | | | | | | | | | |  |

Table 5 Data extraction

| Table 5  Key findings and limitations of studies for the GMA and the predictive ability for CP in late-preterm and term infants with NE | | | | | | | | |
| --- | --- | --- | --- | --- | --- | --- | --- | --- |
| Article | Key findings with respect to GMA and CP | Predictive value of GMA | | | | | Limitations identified by the authors | Summarized reasons for exclusion |
|  |  | Sensitivity (%) | Specificity (%) | PPV | NPV | Other correlations |  |  |
|  |  |  |  |  |  |  |  |  |
|  |  |  |  |  |  |  |  |  |
| Note. CP = Cerebral palsy, GMA = General movements assessment | | | | | | | | |
